# Distinct effects of prematurity on MRI metrics of brain functional connectivity, activity, and structure: Univariate and multivariate analyses

**DOI:** 10.1002/hbm.25456

**Published:** 2021-05-06

**Authors:** Antonio M. Chiarelli, Carlo Sestieri, Riccardo Navarra, Richard G. Wise, Massimo Caulo

**Affiliations:** ^1^ Department of Neuroscience, Imaging, and Clinical Sciences University G. D'Annunzio of Chieti‐Pescara; Institute for Advanced Biomedical Technologies Chieti Italy

**Keywords:** amplitude of low‐frequency fluctuations, functional connectivity, magnetic resonance imaging, multivariate analysis, prematurity, regional volume

## Abstract

Premature birth affects the developmental trajectory of the brain during a period of intense maturation with possible lifelong consequences. To better understand the effect of prematurity on brain structure and function, we performed blood‐oxygen‐level dependent (BOLD) and anatomical magnetic resonance imaging (MRI) at 40 weeks of postmenstrual age on 88 newborns with variable gestational age (GA) at birth and no evident radiological alterations. We extracted measures of resting‐state functional connectivity and activity in a set of 90 cortical and subcortical brain regions through the evaluation of BOLD correlations between regions and of fractional amplitude of low‐frequency fluctuation (fALFF) within regions, respectively. Anatomical information was acquired through the assessment of regional volumes. We performed univariate analyses on each metric to examine the association with GA at birth, the spatial distribution of the effects, and the consistency across metrics. Moreover, a data‐driven multivariate analysis (i.e., Machine Learning) framework exploited the high dimensionality of the data to assess the sensitivity of each metric to the effect of premature birth. Prematurity was associated with bidirectional alterations of functional connectivity and regional volume and, to a lesser extent, of fALFF. Notably, the effects of prematurity on functional connectivity were spatially diffuse, mainly within cortical regions, whereas effects on regional volume and fALFF were more focal, involving subcortical structures. While the two analytical approaches delivered consistent results, the multivariate analysis was more sensitive in capturing the complex pattern of prematurity effects. Future studies might apply multivariate frameworks to identify premature infants at risk of a negative neurodevelopmental outcome.

## Graphical abstract

To better understand the effect of prematurity on brain structure and function, we evaluated BOLD and anatomical MRI metrics in a set of 90 cortical and subcortical brain regions of 88 newborns at 40 weeks of postmenstrual age, with no evident radiological alterations and variable gestational age (GA) at birth. Data‐driven multivariate analyses of these measures were in agreement with univariate findings and indicated that GA at birth was associated with bidirectional and diffuse alterations of BOLD functional connectivity and more focal alterations of regional volume and BOLD activity (assessed through the fractional amplitude of low‐frequency fluctuation, fALFF). Future studies might successfully apply multivariate frameworks to identify premature infants at risk of a negative neurodevelopmental outcome.

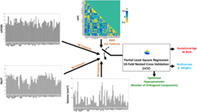

## 1 INTRODUCTION

Recent improvements in neonatal healthcare have reduced the incidence of severe perinatal brain damage and increased survival rates of premature newborns. However, even in the absence of evident brain lesions at standard radiological imaging, this population is still at high risk of a poor neurodevelopmental outcome (Johnson et al., 2009; Mento & Nosarti, 2015). Indeed, the last 3 months of gestational age (GA) are associated with intense brain development, including synaptogenesis, axonal growth, and late neuronal migration (Dehaene‐Lambertz & Spelke, 2015; Gilmore, Knickmeyer, & Gao, 2018; Keunen, Counsell, & Benders, 2017; Ortinau & Neil, 2015). Changes to the developmental trajectory of the brain with premature exposure to the extra‐uterine environment can have profound and long‐lasting consequences (Allen, 2008). Therefore, it is important to identify early markers of alterations of brain development that can be used to guide the treatment of premature infants at a higher risk of poor neurodevelopmental outcomes (Rogers, Lean, Wheelock, & Smyser, 2018).

Advanced magnetic resonance imaging (MRI) techniques have been used for the investigation of developmental alterations in preterm brains (Doria, Arichi, & David Edwards, 2014; Ment, Hirtz, & Hüppi, 2009; Zhang, Shen, & Lin, 2019). Anatomical studies have shown that preterm birth is associated with reduced brain volume, cortical folding, axonal integrity, and microstructural connectivity (Keunen et al., 2016; Lubsen et al., 2011; Volpe, 2009). Studies focusing on functional indices of brain maturation, such as those derived from resting‐state functional connectivity (rsFC) analysis of blood‐oxygen‐level dependent (BOLD) fluctuations (Gao, Lin, Grewen, & Gilmore, 2017; Keunen et al., 2017; Smyser & Neil, 2015), have further revealed the effects of prematurity on the developing connectome, from the reduction of network‐specific connectivity (e.g., Smyser et al., 2010) to whole‐brain network alterations (e.g., Scheinost et al., 2015; Smyser et al., 2016; Smyser & Neil, 2015). Despite its limited use on the infant population, another promising technique to study the effect of prematurity is represented by the amplitude of low‐frequency fluctuations (ALFF, Yu‐Feng et al., 2007; Zou et al., 2008), which indirectly infers the level of local brain activity from the same BOLD data used to estimate connectivity. Although there are reports in literature testing the influence of prematurity on different anatomical metrics derived from clinical MRI (Ball et al., 2017), to our knowledge no study has compared anatomical and functional MRI measures for their association with the degree of prematurity.

Moreover, there is little consensus among studies regarding the localization of prematurity effects. Accumulating evidence highlights a specific impact on subcortical structures and thalamocortical connections, both at the structural (Ball et al., 2012; Ball et al., 2013; Ball et al., 2015) and functional level (Ball et al., 2016; Smyser et al., 2010; Toulmin et al., 2015). These findings are consistent with the notion that the third trimester of gestation is critical for the establishment of functional thalamocortical connections (Kostović et al., 2014). However, other functional studies have reported diffuse group differences, mainly involving cortical networks (Smyser et al., 2016; Smyser, Snyder, et al., 2016). It is not clear whether this apparent discrepancy reflects methodological differences or a distinct a‐priori emphasis on specific brain structures across studies. In addition to the cortical/subcortical distinction, it may also be instructive to examine the relative contribution of short‐ versus long‐range connections and of homotopic versus non‐homotopic connections, given their distinct developmental trajectories described in the literature (Keunen et al., 2017; Ouyang, Kang, Detre, Roberts, & Huang, 2017; Zhang et al., 2019).

The majority of previous studies have traditionally used mass‐univariate testing (Friston, 1994) to investigate the effect of prematurity on MRI metrics. However, finding the link between regional MRI metrics and prematurity can be straightforwardly conceived as a multivariate regression problem (Johnson & Wichern, 2006). Data‐driven multivariate approaches (i.e., Machine Learning) have been recently applied to data from preterm newborns, strongly improving the ability to concurrently correlate multiple neuroimaging features with GA at birth (Ball et al., 2016; Smyser, Dosenbach, et al., 2016). These models can be a‐posteriori analyzed to identify significant variables and can be used for single‐subject inference.

In the present study, we examined the effect of prematurity on measures of resting‐state functional connectivity (rsFC), resting‐state functional connectivity nodal strength (rsFCNS), local activity (fALFF), and regional volume in 90 regions of interest (ROIs) covering the whole brain (Shi et al., 2011). The first aim of the study was to characterize and compare the effect of prematurity on different measures of brain anatomy and function. Thus, we performed region‐based univariate analyses of each metric to explore the association with GA at birth and the spatial consistency across metrics. The second objective was to assess the ability of multivariate analyses to exploit these effects and infer the extent of prematurity. To this aim, we implemented a Machine Learning framework, using partial least square (PLS) regression (Abdi & Williams, 2013) and a cross‐validation scheme (Filzmoser, Liebmann, & Varmuza, 2009).

## 2 METHODS

### 2.1 Population

Infants were recruited from the Neonatology Unit of the University Hospital of Chieti from 2010 to 2018. Neonates underwent standard clinical MRI examination for premature birth or suspected perinatal suffering at the 40th week of postmenstrual age (PMA). Neonates born before 37 weeks of GA were selected based on the following exclusion criteria:

1. Chromosomal abnormality or suspected or proven congenital infection (e.g., HIV, sepsis, toxoplasmosis, rubella, cytomegalovirus, and herpes simplex virus).
2. Neurological abnormalities, including germinal matrix hemorrhage of any grade, cystic periventricular leukomalacia, moderate–severe cerebellar hemorrhage, or lesions in the deep or cortical gray matter.
3. Absent functional MRI.

Neonates born within or after 37 weeks of GA were selected from a group of consecutive neonates without asphyxia that underwent MRI because of periventricular hyperechogenicity at routine early cranial ultrasound. The neonates did not present signal abnormalities at standard MR sequences and had normal neurologic status at a 12‐month clinical follow‐up. The selection resulted in a group of 88 infants born between 25 and 40 weeks (mean = 33 weeks, *SD* = 3.7 weeks), 43/88 patients were female and 15/88 were born at term (>37 weeks of GA at birth). Table 1 summarizes the main demographic and clinical information that were available and their association with GA at birth for the complete population.

**TABLE 1:** Demographic and clinical information

All newborns (*N* = 88)			GA at birth (weeks)		
GA at birth (weeks)—mean (*SD*)	33 (3.75)	Association with GA at birth	25–32 (*N* = 46)	33–36 (*N* = 25)	37–40 (*N* = 17)
PMA at scan (weeks)—mean (*SD*)	40 (0)	‐	40 (0)	40 (0)	40 (0)
Female—*n* (%)	43 (49)	t = 0.34; *p* = n.s.	24 (52)	12 (48)	7 (41)
Multiple gestations—*n* (%)	37 (42)	t = 2.74; *p* < .01	22 (48)	15 (60)	0 (0)
Birth weight at birth (g)—mean (*SD*)	1821 (693)	*r* = .88; *p* < 10^−3^	1,460 (328)	1938 (482)	3,210 (423)
APGAR score at birth—mean (*SD*)	6.4 (2.0)	*r* = .14; *p* = n.s.	6.3 (2.1)	7.4 (1.4)	5.3 (2.3)

The study was part of a clinical radiological screening on premature neonates conducted in the Abruzzo region, Italy, and led by the Local Health Authority. Moreover, the implementation of functional sequences at the end of standard clinical examinations, as well as functional data availability for research purposes, were approved by the Ethics Committee of the University G. D'Annunzio of Chieti‐Pescara. Parental informed consent was obtained for each subject before participation in the study, in accordance with the Declaration of Helsinki and with guidelines set by the Human Studies Committee of G. D'Annunzio Chieti University.

### 2.2 MRI acquisition

MR imaging was performed with a 3 T whole‐body system (Achieva 3.0 T X‐Series) from Philips Healthcare (Best, Netherlands) using an eight‐channel head‐only receiver coil. Participants were fed and then sedated with 0.05 mg oral Midazolam per kilogram of bodyweight immediately before the scans to minimize motion artifacts (Ball et al., 2012; Ball et al., 2016; Stoecklein et al., 2020; Toulmin et al., 2015; van den Heuvel et al., 2015). Neonates were laid in the scanner in a supine position and swaddled in blankets. A molded foam was placed around the body to minimize head movement. Neonatal earmuffs (MiniMuffs; Natus Medical, San Carlos, California) and adapted ear‐canal plugs were used for hearing protection. Heart rate and oxygen saturation were monitored during the MR imaging session by an intensive care neonatologist. Within the standard clinical MRI examination, the structural images used in this study were collected using the T1‐weighted Turbo Field Echo (TFE) sagittal sequence (Flip Angle: 8°; TR: 9 ms; TE: 4.2 ms; voxel size: 1 × 1 × 1 mm^3^; FOV: 200 × 200 × 150 mm^3^) with a whole‐body SAR below 0.2 W/Kg. At the end of standard clinical MRI sequences, whole‐brain functional images were acquired using a T2*‐weighted, echo‐planar imaging (EPI), FFE axial sequence (Flip Angle: 90°; TR: 1555 ms; TE: 30 ms; voxel size: 2.5 × 2.5 × 3 mm^3^; FOV: 180 × 180 × 75 mm^3^; slice gap: 0 mm) with a whole‐body SAR within 0.8 W/kg. The functional scan duration was 4 min and 15 s.

### 2.3 MR image preprocessing

**FIGURE 1:**
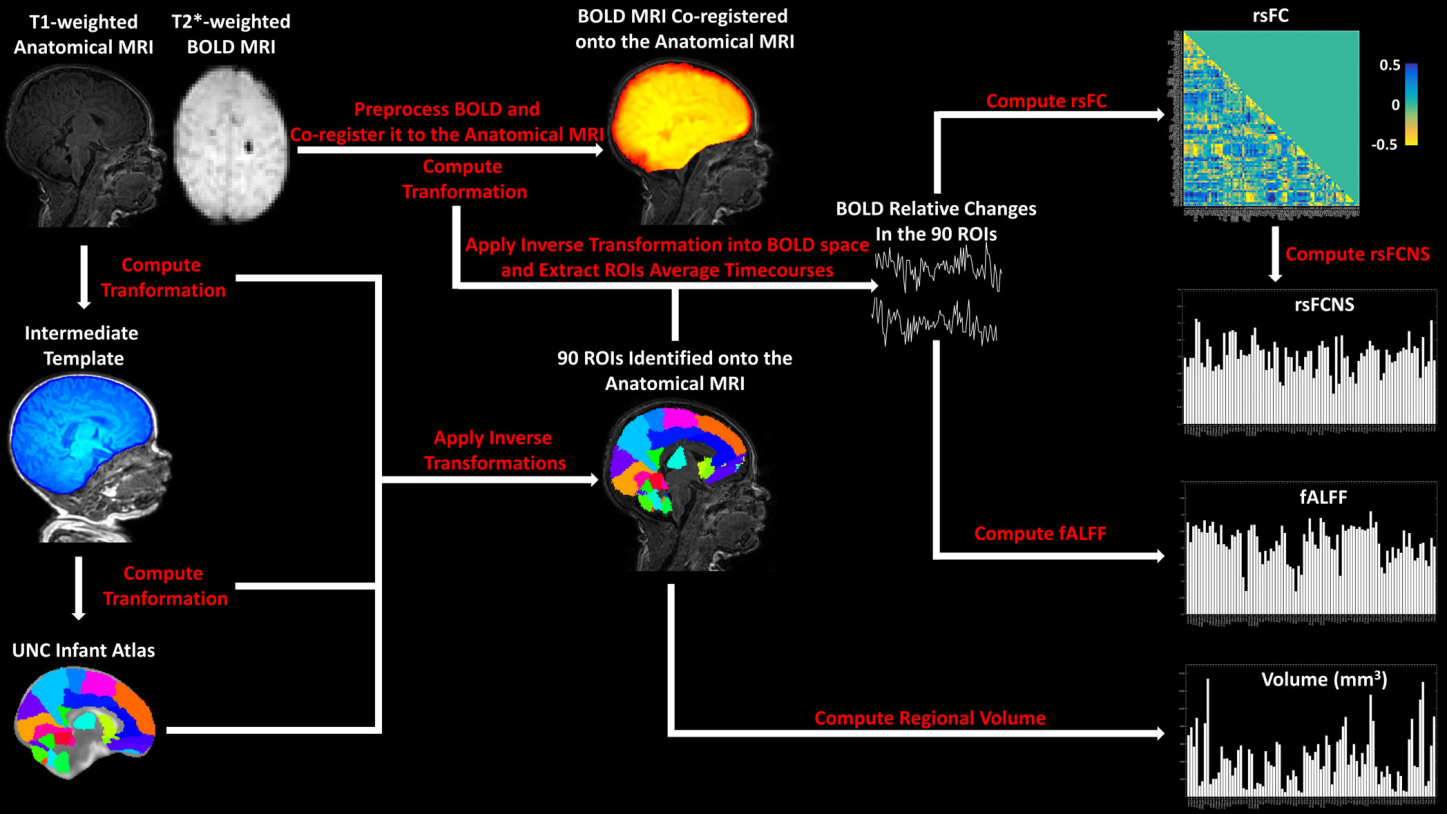
Workflow of the anatomical and BOLD MRI Preprocessing as well as rsFC, rsFCNS, fALFF and Volume computation in the 90 ROIs identified based on the UNC Infant Atlas (Shi et al., 2011)

The MR image processing workflow is reported in Figure 1.

The 90 subcortical and cortical ROIs used in this study were defined based on the University of North Carolina (UNC) Infant Atlas (Shi et al., 2011). Given the difficulties of directly registering each infant anatomical image with the atlas, an intermediate in‐house structural template was built by averaging the infants' T1‐weighted anatomical images (Avants et al., 2010; Avants et al., 2011) and segmenting the brain of the average template by hand (Yushkevich et al., 2006). The T1 in‐house template was built by using the Advanced Normalization Tools (ANTs, http://stnava.github.io/ANTs/) with default settings (Avants et al., 2010; Avants et al., 2011). The segmented brain of the intermediate template was registered to the UNC Infant Atlas. After registering each subject's anatomical image to the in‐house template, inverse transformations into the in‐house template, and then into each structural image, were applied on the UNC Infant Atlas to identify the ROIs in the original subject anatomical space.

EPI T2*‐weighted BOLD images, acquired at rest, were preprocessed according to a standard pipeline (Dolgin, 2010) using a combination of AFNI (Cox, 1996) and FSL (Jenkinson, Beckmann, Behrens, Woolrich, & Smith, 2012) tools. The pipeline included: (a) slice time and motion correction using the 3dTshift and 3dvolreg functions; (b) marking of motion outliers with the fsl_motion_outliers tool using DVARS metric and default settings for the definition of outliers (motion metric above the 75th percentile +1.5 times the interquartile range) (Jenkinson et al., 2012; Power, Barnes, Snyder, Schlaggar, & Petersen, 2012) (c) 4D image scaling using fslmaths (d) linear and quadratic temporal detrending using 3dDetrend (Churchill et al., 2012). The motion parameters (3 translations, 3 rotations, and motion outliers) were finally regressed out (without scrubbing) from the raw BOLD time series, using the tool 3dREMLfit (Bright, Tench, & Murphy, 2017). Finally, the anatomy‐registered atlas was registered to the preprocessed BOLD images, and the average BOLD signal (expressed as relative signal change) was extracted from each ROI identified in the native BOLD space. Of note, all registrations were performed using the state‐of‐the‐art diffeomorphic registration method and the mutual information metric from ANTs (Avants et al., 2010; Avants et al., 2011). The registrations were visually inspected and approved by an expert neuroradiologist.

### 2.4 Functional and structural metrics

#### 2.4.1 Resting‐state functional connectivity

Resting‐state functional connectivity (RsFC) matrices were built by evaluating pairwise associations of BOLD signals in the 90 ROIs while accounting for the contribution of the global signal. Each normalized (z‐scored) ROI's BOLD timecourse was regressed on each other normalized ROI's BOLD timecourse using the normalized average (among the 90 ROIs) BOLD signal as an additional independent variable within a general linear model (GLM) framework (Murphy & Fox, 2017). Note that this GLM analysis is not commutative between ROIs, generating a nonsymmetric connectivity matrix. However, to evaluate undirected connections, the average between the lower and the upper diagonal portions of the connectivity matrices was computed. The latter computational step delivered 4,005 meaningful rsFC connections. An additional control analysis focused exclusively on positive correlations by zeroing out negative correlation values.

#### 2.4.2 Resting state functional connectivity nodal strength

We further evaluated an ROI‐specific metric derived for rsFC depicting the connectivity nodal (i.e., regional) strength. We defined this metric as “resting‐state functional connectivity nodal strength” (rsFCNS). Of note, similar measures have been also referred as “degree centrality” in the literature on graph theory (Holiga et al., 2019; Rubinov & Sporns, 2010).

rsFCNS was evaluated from the rsFC matrices by computing, for the j^th^ ROI, the rsFCNS based on its correlations (rsFC) with the other ROIs according to the formula:

$${\text{rsFCNS}}_{j}=\frac{1}{N-1}\sqrt{\sum_{i=1}^{N}{\left({\text{rsFC}}_{\mathrm{ij}}\right)}^{2}}\;\forall i\neq j$$

where N is equal to the total number of ROIs (i.e., 90).

#### 2.4.3 fALFF

fALFF was evaluated in each of the 90 ROIs of interest using the tool 3dRSFC (Biswal, Kannurpatti, & Rypma, 2007; Yu‐Feng et al., 2007; Zou et al., 2008) and it was computed as the ratio between the BOLD signal power in the 0.01–0.1 Hz frequency range and its total power (from 0.0039 Hz up to the Nyquist frequency of 0.32 Hz).

#### 2.4.4 Volume

Simple metrics of local brain structure can be derived in preterm newborns from standard anatomical images using the deformation field that maps each image to a template and vice‐versa (Ball et al., 2012; Gaser, Nenadic, Buchsbaum, Hazlett, & Buchsbaum, 2001). The exploitation of the deformation field can provide estimates of quantitative regional volume without the need for tissue segmentation, a procedure that is still under development in the newborn population (Makropoulos, Counsell, & Rueckert, 2018). In the present study, we evaluated the volume of the 90 ROIs using the UNC Infant Atlas registered to the individual anatomical images (Ashburner et al., 1998). This analysis provided a quantitative estimate of ROI volumes (expressed in mm^3^) that is directly connected to the extent of regional deformation linking the original template to each subject's anatomy.

### 2.5 Univariate analyses

Univariate analyses were performed on rsFC, rsFCNS, fALFF, and Volume to test their association with GA at birth (expressed in weeks). A first analysis was conducted at a whole‐brain level to evaluate the average rsFC, rsFCNS, and fALFF among ROIs, as well as the total ROIs volume (sum of the individual ROI volumes). Moreover, 4,005 independent rsFC features, each associated with a pairwise correlation between ROIs, as well as 90 rsFCNS, fALFF, and Volume features, each associated with a single ROI, were regressed against GA at birth.

For visualization purposes, and to test the extent of ROI‐based spatial effects of GA at birth, the ROIs were grouped into subcortical (8 ROIs), frontal (34 ROIs), temporal (18 ROIs), parietal (16 ROIs), and occipital (14 ROIs) regions. Cortical ROIs were further subdivided according to their medial or lateral location. Pairwise connections were divided in subcortico‐cortical (N = 688 ) and cortico‐cortical (N = 3,317) connections, long‐range (N = 2002) and short‐range (N = 2003) connections (based on a median split of the distance between their centroids in the UNC Infant Atlas, median value 48.8 mm) and homotopic (N = 45) and non‐homotopic (N = 3,960) connections.

The same univariate analyses on rsFC and rsFCNS were also performed using only positive rsFC correlations (i.e., zeroing out negative correlations). Additional control analyses for the effects of motion were performed on the six DVARS motion signal variances (i.e., the variances of the three translations and the three rotations [yaw, pitch, and roll] along the main axis) and the number of motion outliers in the BOLD acquisition.

### 2.6 Multivariate analyses

**FIGURE 2:**
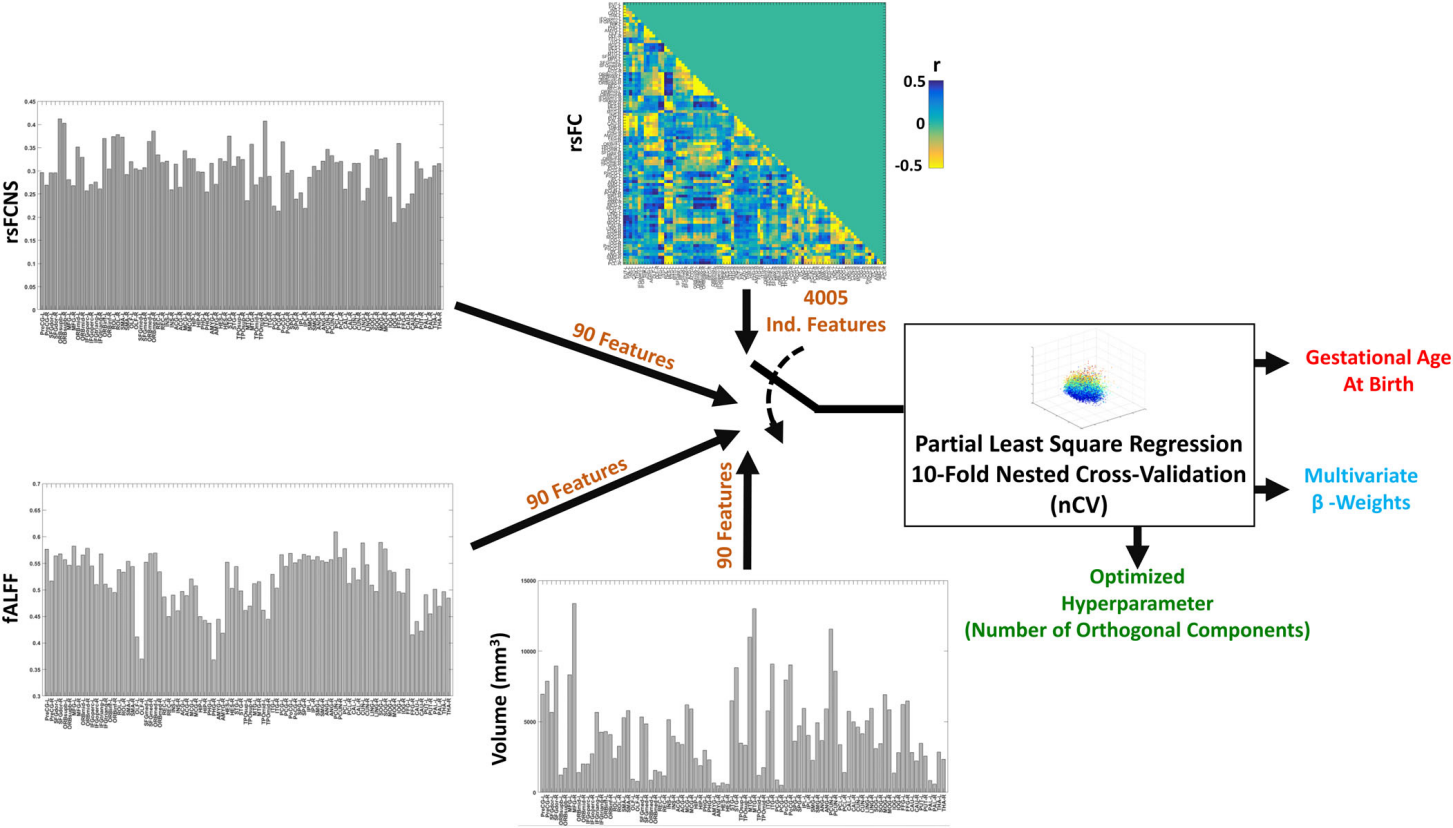
The data‐driven multivariate PLS analyses implemented to infer GA at birth from rsFC, rsFCNS, fALFF and Volume. The optimal number of PLS components, the multivariate β‐weights and the inference performance were estimated through a 10‐fold nCV. The analyses were performed in both a regression and classification (through a median split) modality, and they were also performed on subsamples of the original spaces (e.g., considering only subcortical ROIs)

A data‐driven multivariate analysis framework was implemented to infer GA at birth from different feature spaces (Figure 2). When analyzing the complete data set, the feature spaces were composed of rsFC (4,005 independent variables), rsFCNS, fALLF, or Volume (all the three latter feature spaces composed of 90 independent variables). The number of independent samples was always equal to the number of subjects, that is, 88.

To account for the large numerosity of independent features (e.g., ROIs or ROI connections) (Huopaniemi, Suvitaival, Nikkilä, Orešič, & Kaski, 2009; Kolter & Ng, 2009), we used partial least square (PLS) regression (Wold, Ruhe, Wold, & Dunn, 1984), which reduces the predictors to a smaller set of uncorrelated components maximally associated with the dependent feature/s by exploiting collinearity among independent features (Abdi & Williams, 2013; Chiarelli, Romani, & Merla, 2014). PLS was chosen because of the reduced sample numerosity and the high collinearity among features in the data set. Of note, the learning process (fitting) of the PLS algorithm provides regression loadings that can be used to retrieve the weights (β‐weights) of the original independent variables. To optimize the hyperparameter of the PLS (number of uncorrelated components) and concurrently evaluate the out‐of‐training‐sample performance of the algorithm (i.e., the generalization) (Bishop, 2006), a 10‐fold nested cross‐validation (nCV) was used (Filzmoser et al., 2009; Kearns & Ron, 1999; Krstajic, Buturovic, Leahy, & Thomas, 2014). The number of components allowed during the hyperparameter optimization was constrained between a minimum of 1 and a maximum of 20. The expected β‐weights of the PLS were finally computed by running a single analysis on the complete data set using the rounded average number of components (i.e., the optimal number) delivered by the inner loops of the 10‐fold nCV analysis.

For each feature space, the PLS regression was iterated two times, one in a “regression modality,” where the attempt was to regress GA at birth expressed in weeks, and the other in a “classification modality,” where the PLS was used to classify whether each subject had a GA at birth below or above 32 weeks. This latter analysis was performed by providing to the machinery an output that was either 0 (for a GA below 32 weeks) or 1 (for a GA equal or above 32 weeks).

For rsFC, the 10‐fold nCV analysis was also performed separately on cortical and subcortical connections, on long‐ and short‐range connections, and on homotopic and non‐homotopic connections. For rsFCNS, fALFF, and Volume, the analysis was also performed separately on subcortical and cortical ROIs. To account for pairwise differences in the numerosity of ROIs, or ROI connections, between groups, the multivariate analyses were performed multiple times on the group with the larger numerosity with a random sampling of ROIs to equalize the number of independent features between groups. Average results for this latter analysis are reported in the results section.

In addition, we tested whether combining functional and anatomical information in the inference of GA at birth resulted in a synergistic effect. The rsFCNS was excluded from the analysis since this metric is derived from rsFC and does not encode additional information.

Finally, control multivariate analyses were also selectively conducted on positive rsFC correlations and using motion metrics as input variables.

### 2.7 Statistical inference

Regarding the univariate analyses, ROI‐based regressions of the different metrics with GA at birth were performed and uncorrected, as well as corrected (using false discovery rate (FDR) (Verhoeven, Simonsen, & McIntyre, 2005)), statistical significance was evaluated. To statistically compare the average correlations of rsFC with GA at birth between different groups of ROIs, or ROI connections, a bootstrap approach (Kohavi, 1995) with 10^5^ iterations was used. Furthermore, pairwise correlation analyses of the ROI regression weights between the different metrics were performed to evaluate the extent of similarity in the spatial distribution of the effect of prematurity. For this analysis, both the Fisher‐z transforms of correlation and the β‐weights were used.

Regarding the multivariate analyses in a “regression modality,” the performance was assessed through correlation analyses between the inferred and the true GA at birth. Correlation coefficients were Fisher‐z transformed to treat them as normally distributed and to find statistically significant differences among metrics. Moreover, correlation analyses were performed comparing the univariate and the multivariate regression weights for the different features considering statistical relevance metrics, that is, Fisher‐z transform of correlations or z‐scores, and β‐weights.

Unlike the univariate analysis, the statistical relevance of each multivariate β‐weight could not be computed using closed‐form solutions (e.g., based on the Fisher‐z transform of correlation). Hence, in this case, z‐scores were computed through a bootstrap approach with 10^5^ iterations.

When using the multivariate analysis as a classifier, the performance was assessed through receiver operating characteristic (ROC) analyses. For comparison, the same analyses were also performed on randomly shuffled labels. The ROC analysis delivered an area under the curve (AUC) which was transformed into a z‐score to test its statistical significance.

## 3 RESULTS

### 3.1 MRI preprocessing outcome

**FIGURE 3:**
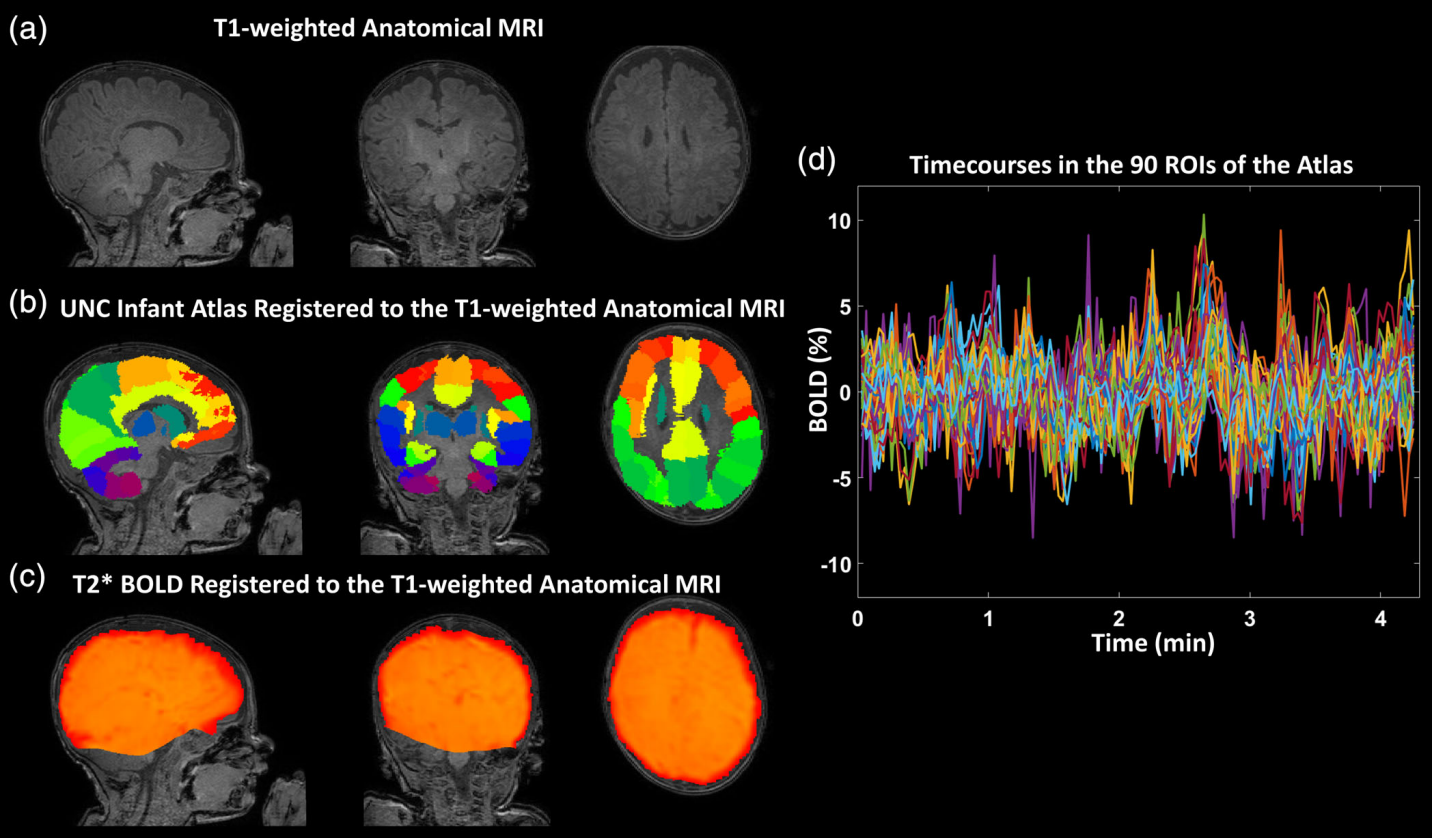
**(**a) Example of a neonate T1‐weighted anatomical MRI. (b) UNC Infant Atlas registered to the T1‐weighted MRI. (c) Motion‐corrected functional BOLD volume registered to the T1‐weighted MRI. (d) 90 preprocessed BOLD signals extracted for the same infant from the ROIs defined in the atlas by transforming the anatomy‐registered atlas into the native BOLD space

Figure 3a–c reports the ANTs registration between T1‐weighted anatomical MRI, UNC Infant Atlas, and a motion‐corrected BOLD volume in an exemplar infant. Figure 3d illustrates the preprocessed BOLD signals extracted from the same infant within the 90 atlas ROIs. The BOLD signals were extracted transforming the anatomy‐registered atlas into the subject native BOLD space. The absence of residual localized motion effects in the BOLD series is evident. Moreover, the amplitudes of BOLD signals of few point percentages are compatible with hemodynamic oscillations and do not appear to reflect a temporally diffuse effect of motion. Both these characteristics are likely due to the mild sedation. As a matter of fact, only an average of seven volumes (*SD* = 5) per subject were deemed as outliers, which where anyhow accounted for during preprocessing.

### 3.2 Univariate analyses

We here report a summary of the results of the univariate analyses. Please refer to File S1 for a more detailed description and the associated figures.

No global effect was found (all *p*'s > .05) and a rather equal amount of positive and negative correlations were observed between regional or inter‐regional metrics and GA at birth.

For rsFC, the results did not suggest strong focal effects (all *r*'s < .45 in modulus). Only two connections with the strongest negative correlations survived multiple comparison corrections. Subcortico‐cortical connections had a significantly lower average correlation with GA at birth compared with cortico‐cortical connections (z = −1.995, *p* = .0231). Moreover, long‐range connections had a significantly higher average correlation with GA at birth compared with short‐range connections (z = 2.336, *p* = 9.8 × 10^−3^).

When collapsing rsFC into rsFCNS, no ROI was significantly correlated with GA at birth after multiple comparison correction (all *r*'s < 0.3 in modulus, all *p*'s = n.s. after FDR). No specific spatial pattern seemed to emerge, except for stronger medial‐frontal connections in more premature infants (i.e., ROIs associated with negative coefficients, z = −1.723, *p* = .0426).

fALFF had weak regional univariate associations with GA at birth (all *r*'s < .2 in modulus, all *p*'s = n.s. after FDR).

Consistent with previous studies looking at the effect of prematurity on regional volume (Ball et al., 2012; Ball et al., 2013; Ball et al., 2015), we found a positive relationship with GA at birth in several subcortical and medial temporal regions. The effect sizes were not large (all *r*'s < .3 in modulus) and none survived multiple comparison correction (all *p*'s = n.s. after FDR). However, when grouping subcortical ROIs, these regions showed increased correlation with GA at birth compared with medial frontal ROIs (z = 1.651, *p* = .0495).

A significant spatial consistency of the effects across metrics was only observed between fALFF and Volume (*r* = .252, *df* = 88; *p* = .0165).

Very similar results were obtained when repeating the rsFC and rsFCNS analyses after zeroing out negative correlations in the rsFC matrix. Moreover, control analysis indicated the absence of a significant correlation between motion metrics and GA at birth (refer to File S1).

### 3.3 Multivariate analyses

**FIGURE 4:**
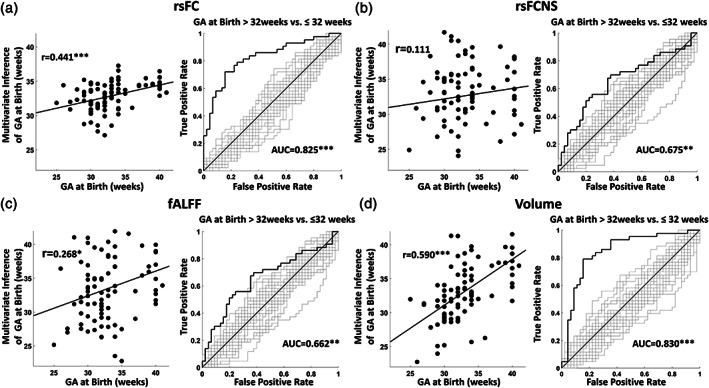
Results of the PLS multivariate analysis on the full set of 90 ROIs. The figure reports the generalization outcomes of the 10‐fold nCV framework for the different metrics of interest. Each panel reports the outcome of the regression (with GA at birth expressed in weeks) and the classification (considering GA at Birth >32 weeks vs. ≤ 32 weeks) approach. The ROC plots true classification curves (black lines) and curves associated with random shuffled data (gray lines). Outcome of the analysis on (a) rsFC, (b) rsFCNS, (c) fALFF, and (d) Volume (* *p* < .05, ** *p* < .01, *** *p* < 10^−3^)

Figure 4 shows the generalization outcomes of the 10‐fold nCV multivariate analyses on the entire data set. The figure illustrates the results obtained with each metric and each panel reports the outcome of the regression (with GA expressed in weeks) and the classification (considering GA at Birth >32 weeks vs. **≤** 32 weeks) approaches. The scatterplot in Figure 4a shows the good results of the multivariate inference of GA at birth using rsFC (*r* = .441, *df* = 86, *p* = 1.7 × 10^−5^, 9 PLS components). The AUC of the classification analysis was 0.825 (z = 5.856, *p* = 2.3·10^−9^, 7 PLS components), in line with previous studies (Ball et al., 2016; Smyser, Dosenbach, et al., 2016). Figure 4b–d reports the results of the same analyses on the regional metrics of rsFCNS, fALFF, and Volume. RsFCNS could not be used to infer GA at birth using a regression modality (*r* = .111, *df* = 86, *p* = n.s., 8 PLS components) but only using a classification modality (AUC = 0.675, z = 3.231, *p* = 6.0 × 10^−4^, 5 PLS components). The analysis of fALFF showed a significant inference of GA at birth (*r* = .268, *df* = 86, *p* = .012, 11 PLS components) and a significant classification performance (AUC = 0.662, z = 2.908, *p* = 1.8 × 10^−3^, 8 PLS components). Volume was the metric that reached the highest performance, both in terms of regression (*r* = .590, *df* = 86, *p* = 1.6 × 10^−9^, 10 PLS components) and classification of GA at birth (AUC = 0.830, z = 7.670, *p* = 8.5 × 10^−15^, 17 PLS components).

**FIGURE 5:**
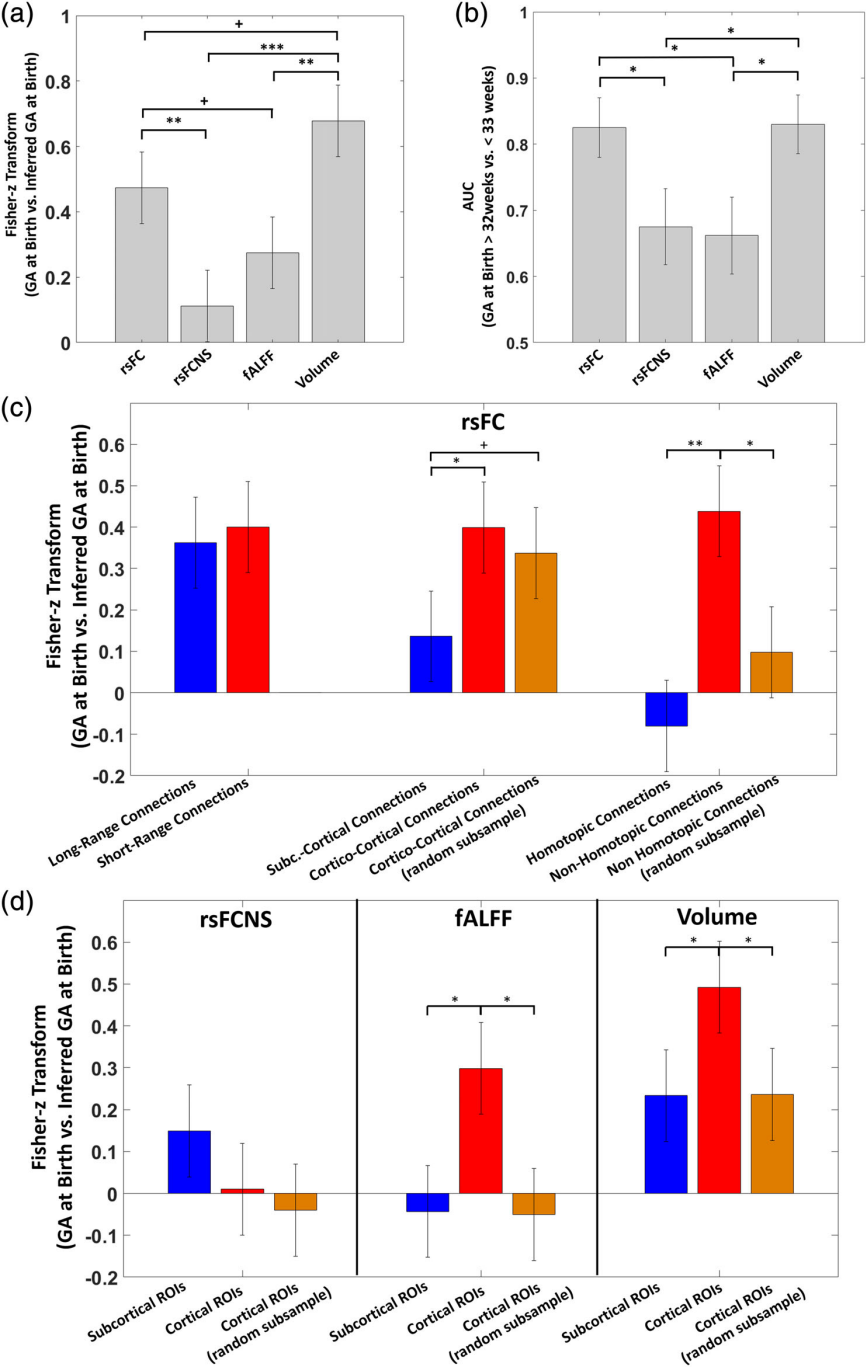
(a) Performance in inferring GA at birth of the 10‐fold nCV PLS multivariate analyses using either rsFC, rsFCNS, fALFF, or Volume in a (a) regression or (b) classification modality. c) Performance and comparison between the 10‐fold nCV analyses conducted in a regression modality of rsFC on GA at birth, when different subsets of rsFC connections were used to compare long‐ versus short‐range, subcortico‐cortical versus cortico‐cortical and homotopic versus non‐homotopic connections. (d) Performance and comparison between nCV analyses conducted in a regression modality of rsFCNS, fALFF and Volume on GA at Birth when subsets of ROIs were used to compare subcortical and cortical ROIs. (^+^ *p* < .10, *p < .05, **p < .01, ***p < 10^–3)^

Figure 5a,b illustrates the results of the direct comparison of the regression and classification performance across metrics. RsFC had better regression performance compared with rsFCNS (z = 2.332, *p* = 9.9 × 10^−3^) and a tendency for a better performance compared with fALFF (z = 1.280, *p* = .10). The regression performance of Volume was significantly higher than that of rsFCNS (z = 3.647, *p* = 1.0 × 10^−4^) and that of fALFF (z = 2.596, *p* = 4.8 × 10^−3^) and it was higher, but only tended toward statistical significance, than that of rsFC (z = 1.315, *p* = .094). The statistical comparison of classification performance indicated a higher performance of rsFC compared with rsFCNS (z = 2.057, *p* = .02) and fALFF (z = 2.219, *p* = .01). Similarly, significantly higher classification performance was observed for Volume over rsFCNS (z = 2.136, *p* = .02) and fALFF (z = 2.298, *p* = .01). No difference was observed between rsFC and Volume and between rsFCNS and fALFF.

We then examined the contribution of different subsamples of ROIs in the inference of GA at birth within the multivariate framework. Figure 5c illustrates the difference in performance in the multivariate inference of GA at birth when using long‐ versus short‐range, subcortico‐cortical versus cortico‐cortical, and homotopic versus non‐homotopic connections in the analysis of rsFC. Supporting the hypothesis of a diffuse effect of premature birth on rsFC, no significant difference was observed for connection length. Interestingly, differences between groups were instead obtained when comparing subcortico‐cortical with cortico‐cortical connections. In this case, a higher regression performance was obtained when considering cortico‐cortical connections, which was statistically significant when using all the available connections (z = 1.693, *p* = .04) and tended toward significance when controlling for the difference in numerosity between the two subgroups (z = 1.307, *p* = .09).

Non‐homotopic connections appeared to perform worse than homotopic connections (z = 3.341, *p* = 4 × 10^−4^). However, the difference did not reach statistical significance when controlling for the lower numerosity of the homotopic connections subgroup. Figure 5d shows the results obtained for the other regional metrics, which were limited to the subcortical versus cortical ROIs comparison. Here, significant larger effects were obtained for cortical connections when considering both fALFF (z = 2.247, *p* = .01) and Volume (z = 1.666, *p* = .04). However, differently from rsFC, these effects vanished when accounting for the larger numerosity of the cortical group. Notably, Volume was the only metric that significantly inferred GA at birth using only subcortical regions (*r* = .229, *p* = .03). No significant difference was found for rsFCNS.

**FIGURE 6:**
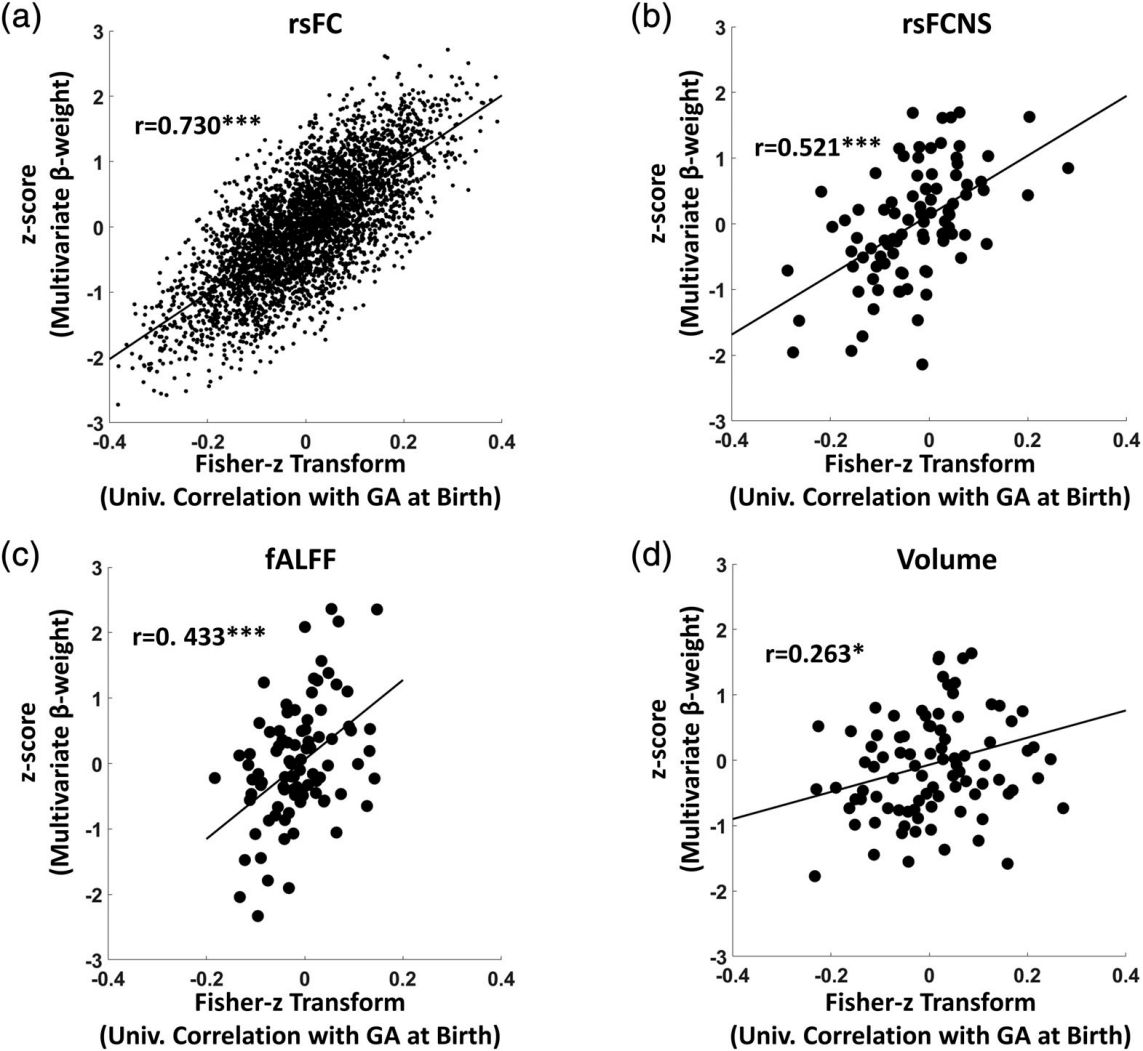
Scatter plots showing the spatial associations between ROIs Fisher‐z transformed univariate correlations and z‐scores of multivariate β‐weights for (a) rsFC, (b) rsFCNS, (c) fALFF, and (d) Volume (*p < .05, ***p < 10^−3^)

As a further investigation of the spatial distribution of the effects of prematurity on the different metrics, we tested for a significant spatial correlation between regression weights obtained with the multivariate and univariate analyses of the whole data set. Figure 6 shows that a robust correlation was observed between multivariate and univariate rsFC weights (Fisher‐z transforms of univariate correlation vs. z‐scores of multivariate β‐weights, *r* = .730, *df* = 4,003, *p* ~ 0), indicating a substantial concordance in the spatial pattern identified by the two approaches (the same analysis performed on the β‐weights is reported in the File S1). A lower, but still highly significant, correlation was found for both rsFCNS (*r* = .521, *df* = 88, *p* = 1.38 × 10^−7^) and fALFF (*r* = .433, *df* = 88, *p* = 3.03 × 10^−5^). Volume exhibited the lowest correlation between multivariate and univariate weights (*r* = .263, *df* = 88, *p* = .012).

The 10‐fold nCV multivariate analysis combining rsFC, fALFF, and Volume resulted in a slightly higher regression (r = .614, *df* = 86, *p* = 2.2 × 10^−10^,10 PLS components) and classification (AUC = 0.846, z = 8.2746, *p* = 1.1 × 10^−16^,19 PLS components) performance compared with individual metrics (refer to File S1 for the related figure), although the increase was no statistically significant (*p*'s > .05).

A control analysis was performed to assess the impact of negative correlations on the results of the analyses on connectivity metrics (rsFC and rsFCNS). When considering only positive correlations, we found a decrease in performance of the multivariate analysis for rsFC and rsFCNS (rsFC with positive correlations only, *r* = .24, *df* = 86, *p* = .02; AUC = 0.72, z = 4.29, *p* = 8.8 × 10^−6^; rsFCNS with positive correlations only, *r* = −.122, *df* = 86, *p* = .25; AUC = 0.47, z = 0.48, *p* = n.s., refer to File S1 for the related figure). However, the decreases were not significant, with a tendency toward statistical significance only for rsFC using the regression modality (z = 1.47, *p* = .07).

Finally, the multivariate analysis using motion information could not significantly infer GA at birth (*p* = n.s.).

## 4 DISCUSSION

The present study examined the effect of premature birth on functional and anatomical metrics of brain development derived from MRI scans, performed at 40 weeks of postmenstrual age (PMA), of 88 infants with gestational age (GA) at birth between 25 and 40 weeks. Note that, since we focused on regression analyses, we mainly treated GA at birth as a continuous variable and we included infants born at term by convention (i.e., after 37 weeks of GA at birth) to increase the numerosity of the sample and the variability of GA at birth. The aim of characterizing the effect of prematurity extent on the brain of newborns thus implies investigating the association of MRI metrics with GA at birth rather than comparing premature infants and infants born at term. To evaluate this association, we exploited the variance of GA at birth in the population under study.

Multivariate analyses better captured the effect of prematurity compared with univariate analyses, albeit the two approaches were generally concordant. In accordance with previous studies, the degree of prematurity was associated with bidirectional alterations of functional connectivity and regional volume. Here, we further demonstrated an association between prematurity and the pattern of fALFF, which is a proxy for regional activity. As expected, relevant volumetric effects were identified in specific regions, such as subcortical structures, and their spatial distribution was similar to that of regional activity. This relative spatial specificity contrasted with the highly diffuse effects observed in functional connectivity, supporting the idea that local structural alterations are associated with a widespread effect on the pattern of connectivity.

### 4.1 Functional connectivity versus local brain function and structure

The multivariate analyses showed that rsFC was the functional metric most sensitive to the effect of prematurity (Figure 4 and 5). Classification performance was comparable to that reported in recent studies adopting similar multivariate approaches (Ball et al., 2016; Smyser, Dosenbach, et al., 2016). Both univariate and multivariate analyses indicated that GA at birth was associated with a complex pattern of increases and decreases of multiple and widespread pairwise connections. Two results strongly support this interpretation: the lack of an effect of GA at birth on mean brain connectivity and the subtle effect on functional connectivity nodal strength, a metric that only provides a general index of how much a region is connected to the rest of the brain. Of note, although the interpretation of negative BOLD correlations is not straightforward, the main results of the study are based on analyses that considered both positive and negative correlations. The results of additional control analyses limited to positive correlations indicated that negative correlations add information to the multivariate framework, supporting previous evidence for the functional significance of anticorrelations in healthy subjects (Kelly, Uddin, Biswal, Castellanos, & Milham, 2008) and neurological patients (Baldassarre et al., 2014). Although the issue is still debated, some authors have proposed that positive and negative correlations might represent a signature of functional integration and segregation, respectively. For example, Baldassarre and colleagues have demonstrated in multiple studies (reviewed in (Baldassarre, Ramsey, Siegel, Shulman, & Corbetta, 2016)) that the behavioral deficits following stroke are associated with both reduced interhemispheric functional connectivity and reduced anti‐correlation between the frontoparietal and the default mode networks. The authors interpreted the former effect as a loss of functional integration within function‐specific networks and the latter effect as a loss of segregation between networks that generally show opposite task‐evoked BOLD activity. Regardless of the specific interpretation given to negative correlations, the results of the present study indicate the significance of this phenomenon for the functional development, which deserves further investigations. Overall, the present results provide support for the crucial role of establishing, but also balancing, the strength of specific functional connections in this phase of brain development (Smyser & Neil, 2015; Zhang et al., 2019).

Convergent evidence about the long‐lasting effects of prematurity on resting‐state (e.g., Constable 2013) and task‐evoked functional connectivity (e.g., Myers 2010) has led to the hypothesis that neurocognitive disorders associated with preterm birth might represent a disease of brain connectivity (Lubsen et al., 2011). However, we stress that the present direct comparison between different metrics did not show a larger effect of GA at birth on measures of connectivity compared with regional volume (Figure 5a,b). Indeed, the best multivariate predictor of gestational age was regional volume, although the difference with functional connectivity was not statistically significant. This result is remarkable, considering that volume was inferred in a rather coarse manner through deformation fields to a common atlas (Gaser et al., 2001). We suggest that even better performance might be achieved using brain segmentation algorithms (Fischl et al., 2002) which, however, are still under development for this population (Makropoulos et al., 2018).

To our knowledge, this is the first study to show that GA at birth can be inferred at 40 weeks of PMA using measures of regional activity (i.e., fALFF). This result extends the findings of a recent study (Shang 2019) that reported a similar classification performance using fALFF in adults born preterm. Here we further demonstrated that regional *negative* associations between activity and GA at birth (greater activity in early preterm infants), which has been previously thought to reflect compensatory effects taking place in later phases of brain development (e.g., Shang 2019, see also Karolis 2017 for results on regional volume), can already be detected around birth, simultaneously with positive effects (greater activity in late preterm) in other regions. It remains to be determined if increased regional activity or volume in premature infants reflects a compensatory phenomenon or the failure of regulatory mechanisms acting on regional development.

Overall, we believe that the present findings support the view that premature birth manifests as a complex interaction of processes taking place within and between tissue compartments and brain regions (Volpe, 2009) and going beyond what can be described considering single metrics.

#### 4.1.1 Focal versus diffuse effects of prematurity on brain function and structure

Consistent with previous work (Ball et al., 2012), both the univariate and the multivariate analyses indicated that volume reduction in premature infants occurred, albeit not exclusively, in subcortical regions (Figure 5d and File S1). Subcortical volume reduction is thought to reflect the vulnerability of deep gray matter to the risks of the extra‐uterine environment (Boardman 2006; Srinivasan 2007) and to further initiate a cascade of functional and structural brain alterations in allied brain structures (Ball et al., 2012). Of note, regional volume exhibited the lowest spatial correlation between multivariate and univariate weights (Figure 6d), suggesting that the multivariate analysis exploited interregional dependencies of volume changes with GA at birth that could not be captured by the univariate analysis.

A spatial consistency of the effect of GA at birth was found between Volume and fALFF in both univariate (refer to Univariate Analysis Results and File S1) and multivariate (Figure 5d) analyses, and this is likely explained by the effect of structural alterations on local physiology. Previous studies in adults born preterm indicate that premature birth causes an opposite pattern of volume reduction in medial temporal regions and volume increase in frontal and lateral temporoparietal cortices (Karolis et al., 2017; Nosarti et al., 2011; Scheinost et al., 2015). Here we found partially consistent results, by showing lower regional activity within medial temporal structures and increased volume in several medial frontal regions in earlier preterms, although their relationship with GA at birth was not particularly robust (refer to Univariate Analysis Results and File S1).

In contrast to regional metrics, functional connectivity appeared to be diffusely altered by prematurity. First, the connectivity nodal strength could not predict GA at birth in the multivariate analysis (Figure 4b), indicating the limited importance of individual nodes. Second, univariate analyses did not identify large differences in the association with GA at birth when comparing long‐ versus short‐range, homotopic versus non‐homotopic, and subcortico‐cortical versus cortico‐cortical connections. Finally, the multivariate analyses identified a tendency toward a larger involvement of cortico‐cortical connections compared with subcortico‐cortical connections. Therefore, prematurity did not have a selective effect on connections that typically show earlier development (i.e., homotopic, short‐range, subcortico‐cortical, reviewed in (Keunen et al., 2017; Ouyang et al., 2017; Zhang et al., 2019)) nor on subcortico‐cortical connections that are thought to have a driving role in the formation of emerging cortical circuits (Ball et al., 2016).

The existing literature on the spatial extent of the effect of prematurity is debated. On the one hand, some studies have demonstrated a predominant reduction of subcortico‐cortical functional connectivity in preterm newborns (Ball et al., 2016; Smyser et al., 2010; Toulmin et al., 2015), similarly to the focal effects observed for regional volume (Ball et al., 2012; Boardman et al., 2006) and anatomical connectivity (Ball et al., 2013; Ball et al., 2015). On the other hand, other studies have clearly shown that the effects of prematurity are instead more complex and diffuse (Smyser & Neil, 2015; Smyser, Snyder, et al., 2016). The results of the present study strongly support the second view and further point to the advantage of multivariate analyses in detecting such complex and distributed patterns of functional connectivity alterations. Concerning this issue, the multivariate analyses comparing groups of ROIs (Figure 5c,d) indicated that the present results were not driven by the PLS algorithm, which might emphasize non‐sparse solutions. In fact, despite multivariate algorithms generally decrease generalization performance as a function of the number of independent variables (i.e., overfitting phenomenon), in our case we observed an increase in the accuracy of the inference as a function of the number of ROIs included in the analysis, consistent with a true widespread effect.

Accumulating evidence from research on stroke patients strongly supports the view that focal brain lesions can induce changes of functional connectivity well beyond the site of damage (Baldassarre et al., 2016, Bayrak 2019, DeMarco 2020), findings that are supported by computational models (e.g., Alstott 2009). In a similar vein, we propose that more focal structural and functional damage to specific brain regions such as the thalamus or the basal ganglia, which are particularly sensitive to premature extrauterine exposure, can result in the widespread imbalance (increase or decrease) of several neocortical connections, due to the number of structural and functional links between these structures and the neocortex. Future studies might combine multivariate approaches with graph theory to better investigate this issue, given the capability of graph theory metrics to summarize global properties of brain networks (Cao et al., 2017; Scheinost et al., 2016).

#### 4.1.2 Univariate versus multivariate analysis

Whereas univariate and multivariate analyses yielded spatially consistent results, the multivariate analysis proved to be more sensitive to the effect of prematurity. The advantage of multivariate analysis likely reflects the ability to analyze a large data set all at once, capturing relative dependencies among variables. The limited number of PLS components identified in our study indicated the ability of the multivariate algorithm to capture shared effects among regions.

However, a drawback of multivariate approaches is that the identified patterns (i.e., the model weights) cannot be straightforwardly interpreted as in the case of univariate analyses, making it difficult to make inferences on the direction of the identified relationships (Carvalho, Pereira, & Cardoso, 2019). Because of the limited interpretability of the multivariate weights, we limited our investigation to the spatial comparison with the univariate weights. Indeed, we found a strong correlation between univariate and multivariate weights across the different metrics, with the notable exception of regional volume. This result suggests that, at least for volume, the high multivariate regression performance exploits inter‐regional effects (e.g., a significant difference in the rate of volumetric growth between two regions). Further studies should be aimed at shedding light on this aspect.

#### 4.1.3 Main limitations and future perspectives

A limitation of the present study was the use of a short BOLD acquisition time, driven by the limited time available for conducting a relatively nonclinical evaluation in a clinical environment. To maximize the efficacy of the standard clinical assessment, newborns were also mildly sedated using Midazolam, which might have altered brain activity and hemodynamics. Future studies should therefore replicate the present findings using a longer acquisition time and alternative or no sedation approaches. However, longer acquisition times are not expected to modify, but only to expand, our positive findings. Moreover, the newborns in our study were sedated with a Midazolam dosage proportional to their weight. Furthermore, the study did not investigate average functional indices, which are influenced by sedation, but focused on the cross‐sectional differences among newborns as a function of GA at birth. It is therefore unlikely that the observed differences between newborns with varying GA at birth merely reflect a confounding effect of sedation, since this possibility would require that the effect of Midazolam per unit‐weight, when administered at the same PMA, changes as a function of GA at birth, an effect that has not been described in the literature. Even in the presence of residual subject‐specific effects, the disadvantage of small alteration in brain hemodynamics should be over‐compensated by the large reduction in motion artifacts. Another limitation of the present retrospective study was the reduced number of clinical information available for all the infants. Although we explicitly removed from the study infants with evident alterations at standard radiological assessment, and we found no relationship between the main clinical variable that was available for all the newborns, the APGAR score soon after birth (Finster, Wood, & Raja, 2005), and the extent of prematurity, we cannot definitively rule out the presence of subtle clinical confounders.

Research about the effect of prematurity on brain development has the ultimate goal of identifying infants at higher risk of a negative outcome (Rogers et al., 2018). In this respect, the lack of a neurocognitive evaluation represents a limitation of the present study. We note, however, that the identification of early biomarkers of an adverse neurodevelopmental outcome is not straightforward. For example, limiting the neurocognitive assessment to the first 1 or 2 years of life, the typical range of the majority of the studies, might not be effective in detecting cognitive deficits that can manifest later in life, that is, at school age and beyond (Mento & Nosarti, 2015). The design of long‐term studies is, therefore, a priority for future investigations. Indeed, data‐driven multivariate models could in theory be used beyond inference of GA at birth for predicting the long‐term neurodevelopmental outcome based on functional and structural brain data and to inform personalized therapy strategies based on those inferences. One can imagine a virtuous cycle in which clinical outcomes are used to continuously improve the predictive ability of such a neuroimaging framework once it enters clinical practice.

## 5 CONCLUSION

The present study demonstrated that prematurity is associated with a complex pattern of bidirectional alterations of MRI‐derived BOLD functional connectivity and regional brain volume, and, to a lesser extent, with modification of fALFF. The analysis of the spatial distribution of the effects indicated that structural alterations, which tend to localize to subcortical structures, can have a widespread effect on functional connectivity between cortical structures. The higher sensitivity of multivariate approaches in identifying the complex effects of GA at birth on multiple MRI metrics suggests their use in future studies for the prediction of neurodevelopmental outcome based on the observed neuroimaging alterations.

## CONFLICT OF INTERESTS

The authors declare no competing financial and non‐financial interests.

## Supporting information


**Appendix** **S1.** Supporting Information.

## Data Availability

The data of the present study are publicly available, in Matlab format, at: https://figshare.com/articles/dataset/Shared_Data_rsFC_Premature_UdA_7z/14178956
