# Supplementary material for: Distinct effects of prematurity on MRI metrics of brain functional connectivity, activity, and structure: Univariate and multivariate analyses
Source: Hum Brain Mapp. 2021 May 6;42(11):3593–607. doi: 10.1002/hbm.25456 (PMC8249887; doi:10.1002/hbm.25456)
Supplement: Supplementary file 1 — Appendix S1. Supporting Information. [file HBM-42-3593-s001.pdf]

Distinct Effects of Prematurity on MRI Metrics of Brain Functional Connectivity, Activity and Structure: Univariate and Multivariate Analyses

Chiarelli A.M.*, Sestieri C.*, Navarra R., Wise R.G., Caulo M.

University G. D'Annunzio of Chieti-Pescara, Department of Neuroscience, Imaging, and Clinical Sciences; Institute for Advanced Biomedical Technologies, Via Luigi Polacchi 13, Chieti, Italy, 66100

**The authors contributed equally to the study.*

Corresponding Author:

Dr. Antonio Maria Chiarelli Ph.D.

University 'G. d'Annunzio' of Chieti-Pescara

Institute for Advanced Biomedical Technologies

Via Luigi Polacchi 13, Chieti, Italy, 66100

Email: antonio.chiarelli@unich.it

Supplementary Information

SI1. MRI Pre-processing (Additional Information)

Figure SI1 reports the outcome of the ANTs registration between the UNC Infant Atlas and the in-house T1-weighted anatomical template. The in-house template was used within an intermediate step for registering the atlas to the T1-weighted anatomical MRI of the infants.

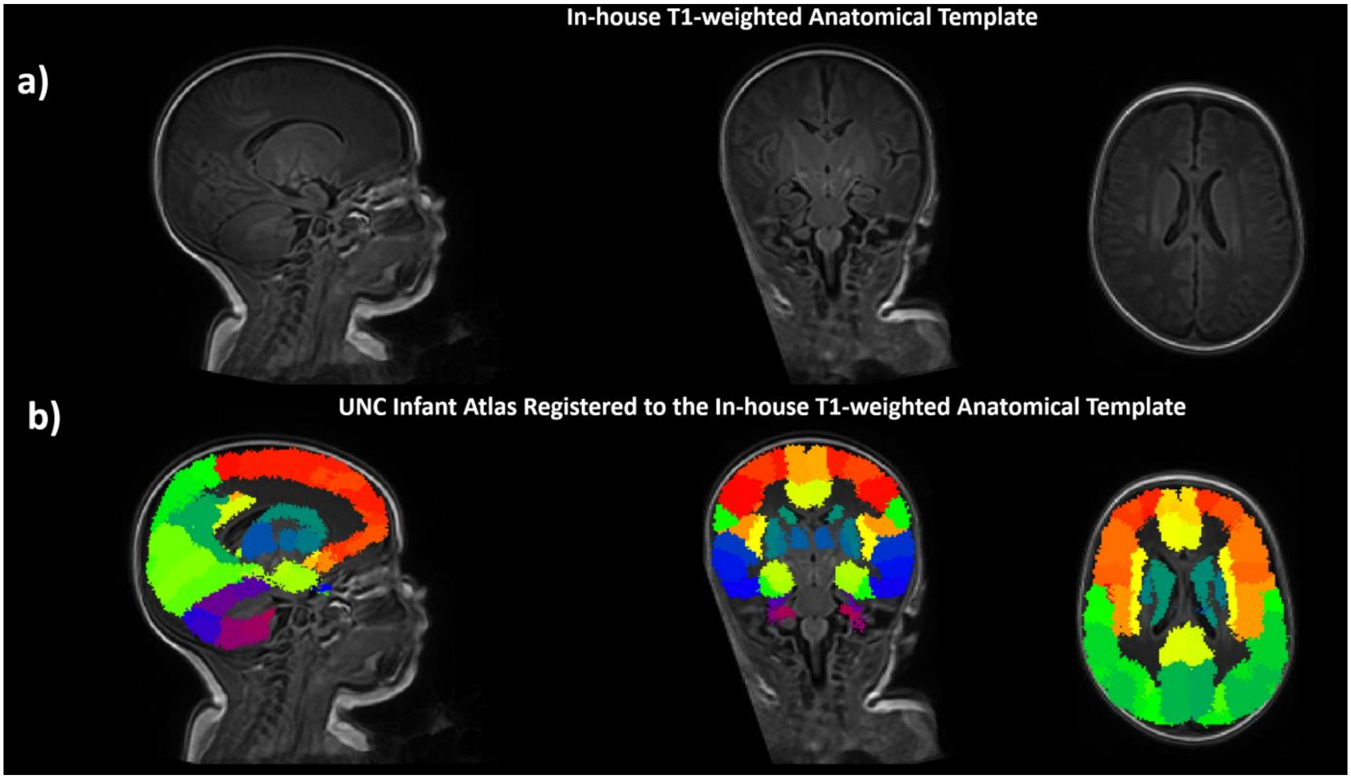

Figure SI1: a) In-house T1-weighted anatomical template used in the study. b) Outcome of the ANTs registration between the UNC Infant Atlas and the in-house anatomical template.

SI2. Results of the Univariate Analyses (Extended)

The association between the average index of rsFC (across subjects mean=-0.012; SD=0.195) and GA at birth was not significant (r=-0.03, df=86; p=n.s.) suggesting that prematurity does not have a net global effect on the functional connectome. Similar correlations with GA at birth were obtained for indices of average rsFCNS (r=-0.04; df=86; p=n.s.), average fALFF (r=-0.02; df=86; p=n.s.), and total Volume

(r=-0.02, df=86; p=n.s.). These findings suggest that the effect of prematurity, if present, had a strong spatial dependence, which could be driven by highly localized or diffuse heterogeneous effects.

To explore the spatial distribution of the effects, we tested for a univariate association between GA at birth and each pairwise index of rsFC. The results of the analyses are shown in Figure SI2 and SI3. Figure SI2a reports the correlation coefficients of the association between each connection of the rsFC matrices with GA at birth. To help the interpretation of the results, Figure SI2b illustrates the 90 inter-regional connections showing the largest positive (left) and negative (right) association with GA at birth, sorted by their correlation coefficient in descending order. Figure SI3 reports the corresponding β-weights of the correlation coefficients reported in Figure SI2. The 90 ROIs included in the analysis are grouped by lobe membership and location on the mediolateral axis. The qualitative impression from figure SI2 is that the effect sizes are not particularly large, reaching approximately 0.4 of maximum correlation magnitude in both positive and negative directions. While several connections reached uncorrected statistical significance, only the two connections with the strongest negative correlations survived multiple comparison corrections. In general, the results did not suggest the presence of strong focal effects induced by prematurity.

We found that subcortico-cortical connections had significantly lower average correlation with GA at birth compared to cortico-cortical connections (z =-1.9953, p=0.0231). Interestingly, long-range connections had significantly higher average correlation with GA at birth compared to short-range connections (z=2.3357, $p=9.8\cdot10^{-3}$). No statistically significant difference was found when comparing homotopic vs. non-homotopic average connections (z=0.1389, p=n.s.). Notably the effect sizes of these statistically significant comparisons were particularly small, and they were only significant when performing pairwise comparison and not when evaluating the departure of each average correlation from 0.

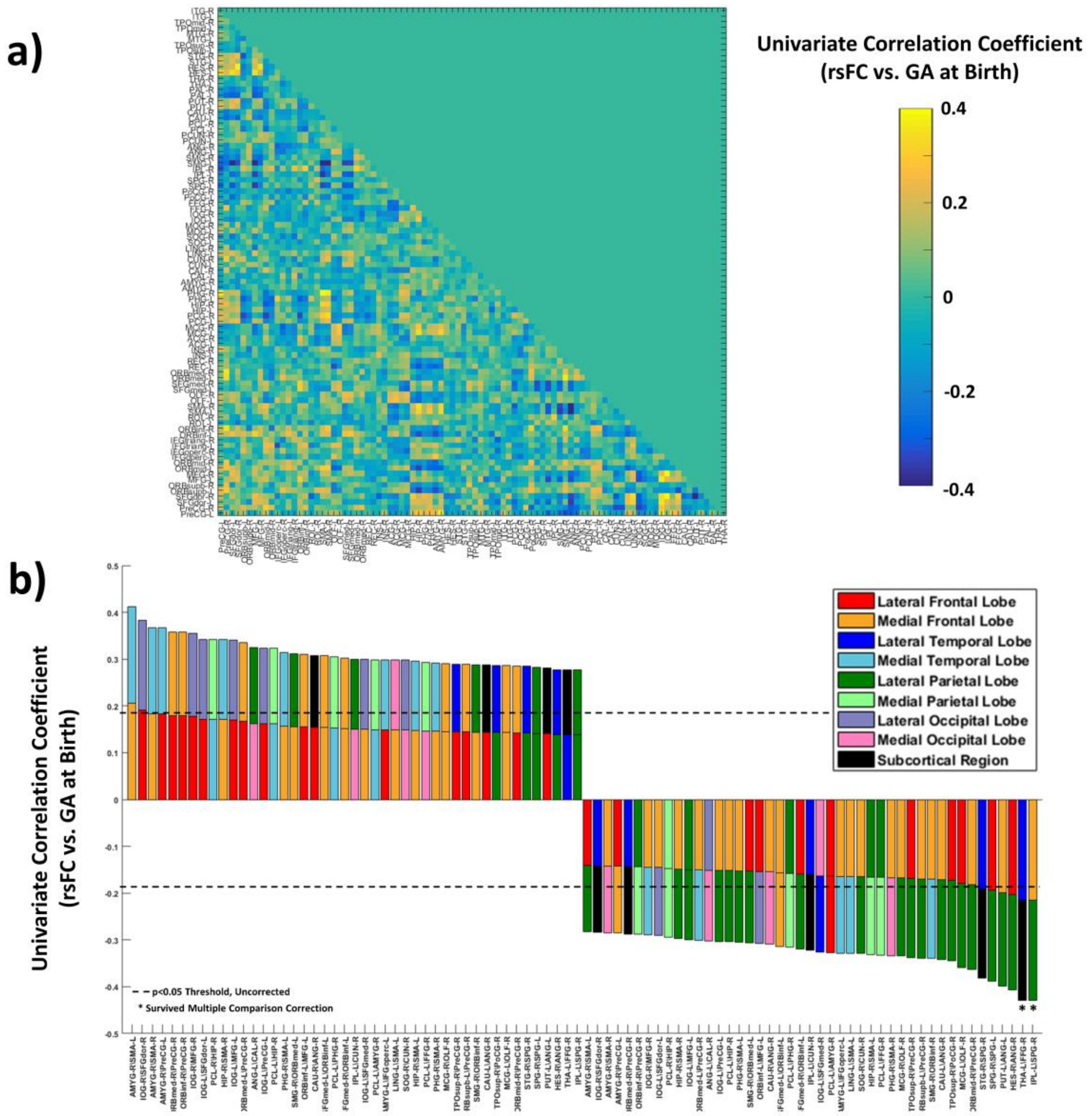

Figure SI2: a) Correlation coefficients of the univariate analyses linking each connection of the rsFC matrices with GA at birth. b) 90 pairwise connections showing the largest positive (left) and negative (right) association with GA at birth, ordered by their correlation coefficient. The 90 original ROIs are grouped by lobe membership and location on the mediolateral axis.

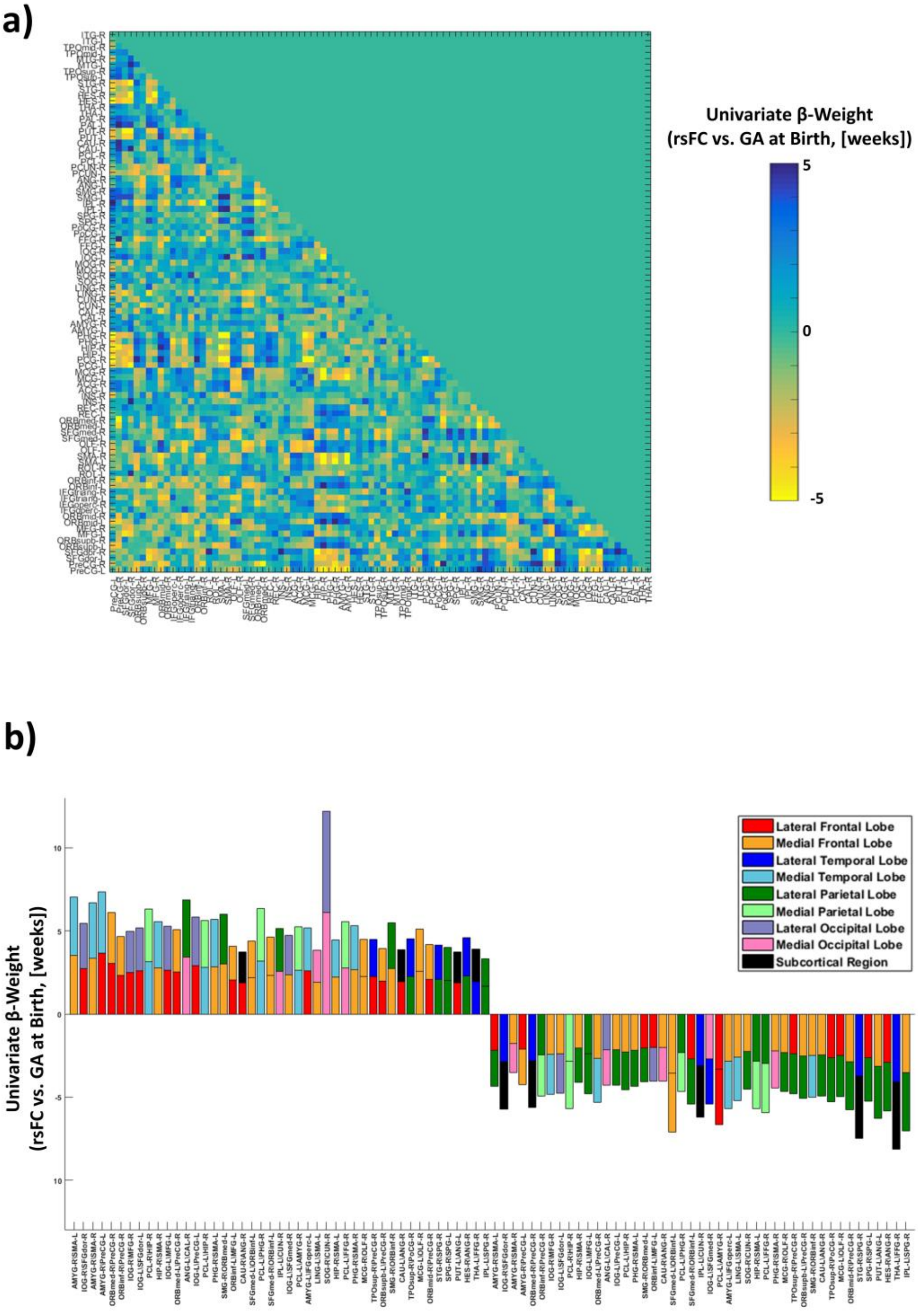

Figure SI3: a) β-weights of the univariate analyses linking each connection of the rsFC matrices with GA at birth. b) β-weights corresponding to the 90 inter-regional connections showing the largest positive (left) and negative (right) correlation with GA at birth, ordered by their correlation coefficient. The 90 original ROIs are grouped by lobe membership and location on the mediolateral axis.

Figure SI4 reports the correlation coefficients of the univariate associations between GA at birth and rsFCNS, fALFF and Volume in the 90 ROIs considered (the corresponding β-weights are presented in Figure SI5).

Figure SI4a shows that, when rsFC was collapsed on the region-based rsFCNS metric, the correlations with GA were not strong (only 8 regions reached the uncorrected significance threshold, and none survived correction for multiple comparisons). Moreover, the results provided further support for the lack of a functional alteration of subcortical connectivity in prematurity, as none of the connections involving subcortical structures reached statistical significance and no significant difference was found when comparing subcortical vs. cortical average rsFCNS correlation with GA at birth (z=0.075, p=n.s.). In general, no specific spatial pattern seemed to emerge, except for stronger medial-frontal connections (orange bars) in more premature infants (i.e. ROIs associated with negative coefficients, z=-1.7226, p=0.0426).

Figure SI4b reports the ordered correlation coefficients concerning the association between fALFF and GA at birth. Notably, only one correlation reached the uncorrected statistical significance threshold but did not survive multiple comparisons correction. In general, this measure showed a weaker association (all correlation coefficient magnitudes <0.2) with GA at birth compared to measures of connectivity. However, a closer inspection of the regions showing the highest positive association with GA at birth revealed the consistent presence of both subcortical (black bars) and medial temporal (cyan bars) regions. While these findings appear consistent with the structural effects of prematurity on these regions already reported in the literature, the effects did not reach statistical significance for either set of ROIs (subcortical, z=-0.4647, p=n.s.; medial temporal, z=1.09, p=n.s.).

In line with the literature [Ball et al., 2012; Ball et al., 2013; Ball et al., 2015], we found a significant positive relationship between Volume of several subcortical and medial temporal regions and GA at

birth. Specifically, Figure SI4c shows that all the subcortical (with the exception of the left caudate), and the medial temporal (with the exception of the left parahippocampal gyrus) ROIs were positively associated with GA at birth, whereas the majority of frontal regions showed a negative association. Again, the effect sizes were not particularly large (all correlation coefficient magnitudes <0.3). Only a few correlations reached uncorrected statistical significance (11 ROIs) but none survived multiple comparison correction. Notably, three of the four regions showing significant negative association with GA at birth were in the medial frontal lobe, in apparent consistency with the rsFCNS results. A statistical assessment confirmed that subcortical ROIs, although not statistically different from 0, had more positive average correlation with GA at birth compared to medial frontal ROIs ($z=1.651$, $p=0.0495$).

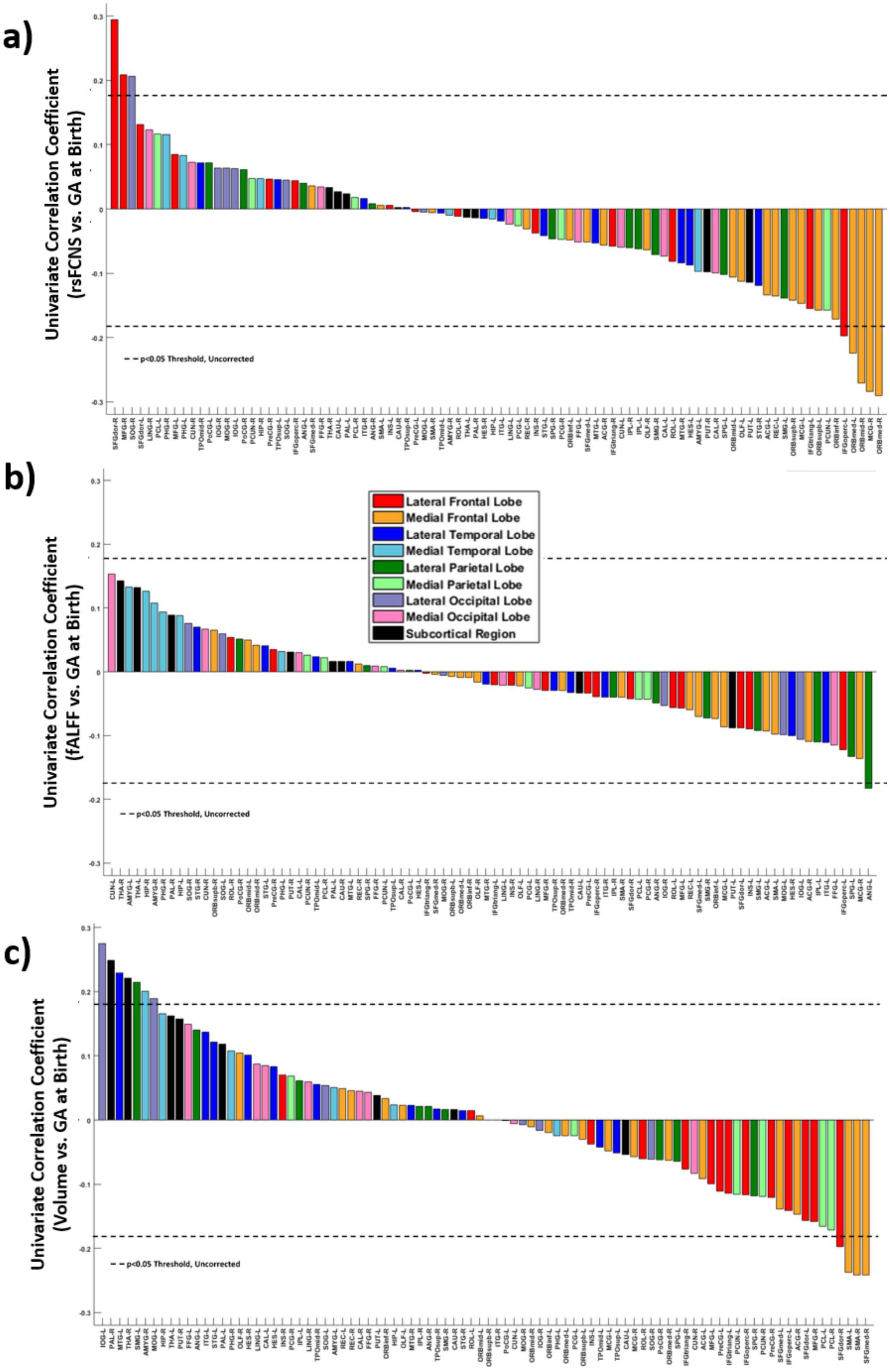

Figure SI4: a) Correlation coefficients of the univariate analyses linking regional metrics in each of the 90 ROIs considered with GA at birth (the corresponding β-weights are reported in the Supplementary Information), sorted as a function of their magnitude and sign in a descending order. Correlation coefficients for a) rsFCNS, b) fALFF and c) Volume.

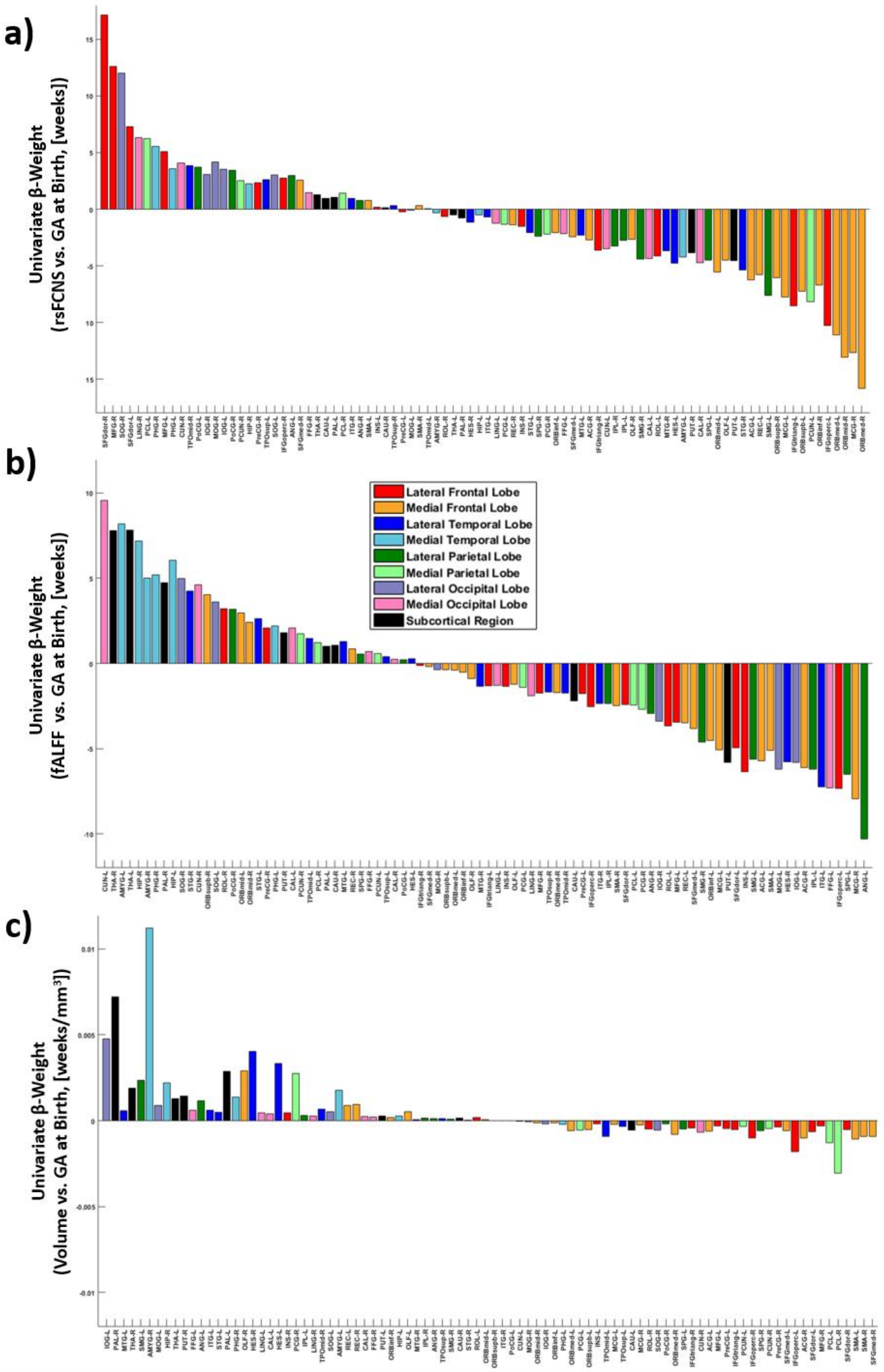

Figure SI5: a) β-weights of the univariate analyses linking regional metrics in each of the 90 ROIs considered with GA at birth, sorted as a function of correlation coefficient magnitude and sign in a descending order. β-weights for a) rsFCNS, b), fALFF and c) Volume.

To assess the spatial consistency of the effects among different MRI metrics, we tested for a significant pairwise relationship between the Fisher-z transformed correlation coefficients of GA at birth of i. regional functional connectivity, ii. local activity and iii. Volume (Figure SI6, the results of the same analysis using the β-weights are reported in Figure SI7). We limited the analysis involving the connectivity to the rsFCNS, given its more direct spatial comparability to fALFF and Volume. As shown in Figure SI6, significant spatial consistency in the 90 ROIs considered was observed between the fALFF and Volume ($r=0.252$, $df=88$; $p=0.0165$, Figure SI6c), whereas no significant correlation involving measures of rsFCNS was observed (Figure SI6a,b).

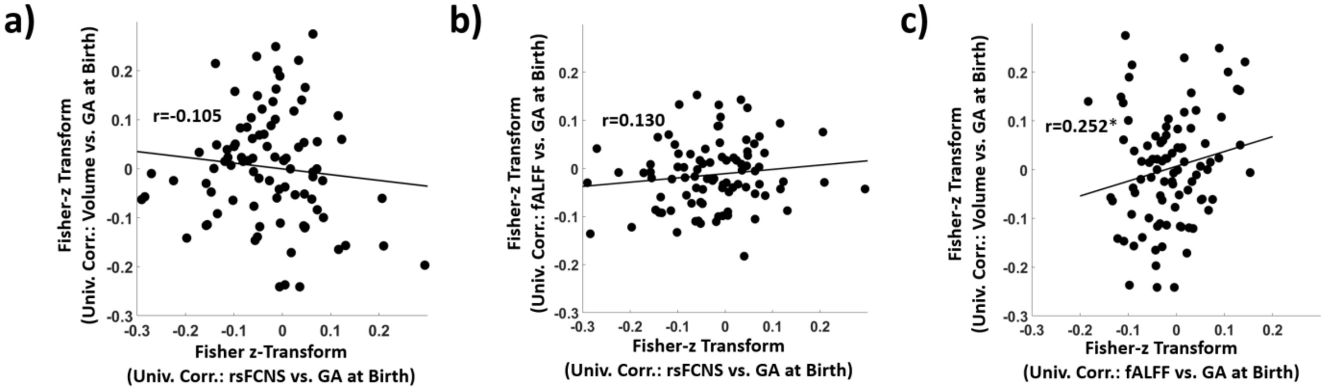

Figure SI6: Scatterplots depicting the pairwise spatial associations between the effects in the 90 ROIs of GA at birth on the regional metrics (expressed as Fisher-z transform of univariate correlations for a) rsFCNS vs. Volume, b) rsFCNS vs. fALFF and c) fALFF vs. Volume (* $p<0.05$).

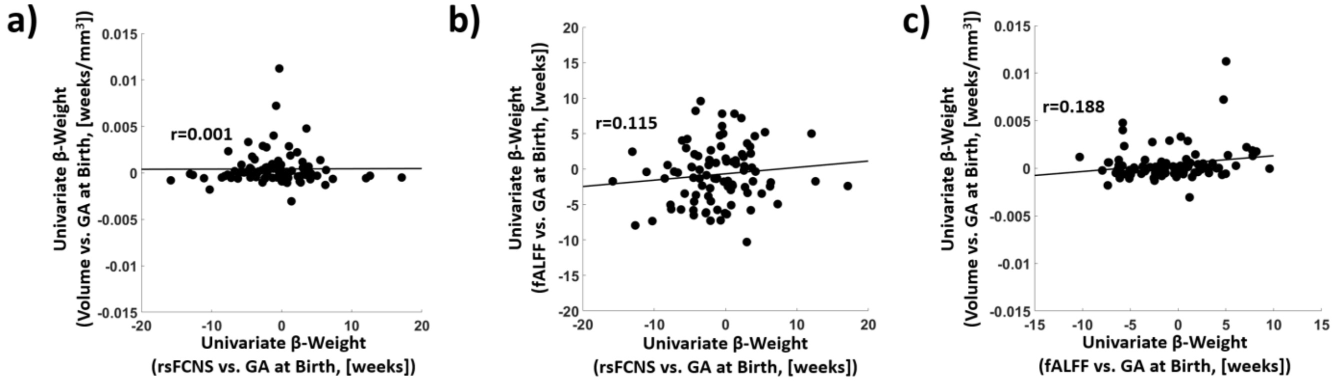

Figure SI7: a) Scatterplots depicting the pairwise spatial associations between the effects in the 90 ROIs of GA at birth on the regional metrics (expressed as β-weights) for a) rsFCNS vs. regional volume, b) rsFCNS vs. fALFF and c) fALFF vs. Volume.

Finally univariate control analyses were performed.

Firstly we evaluated the univariate correlations of rsFC and rsFCNS, when zeroing out negative correlations in the rsFC matrix and compared them with the original rsFC and rsFCNS. Figure SI8 shows the results. We obtained very similar patterns between the two approaches with a comparable magnitude of the correlations with GA at Birth and with a high spatial association with and without zeroing out negative correlations (Figure SI8; rsFC, r=0.85, df=4003, p=0; rsFCNS, r=0.93, df=88, p=0).

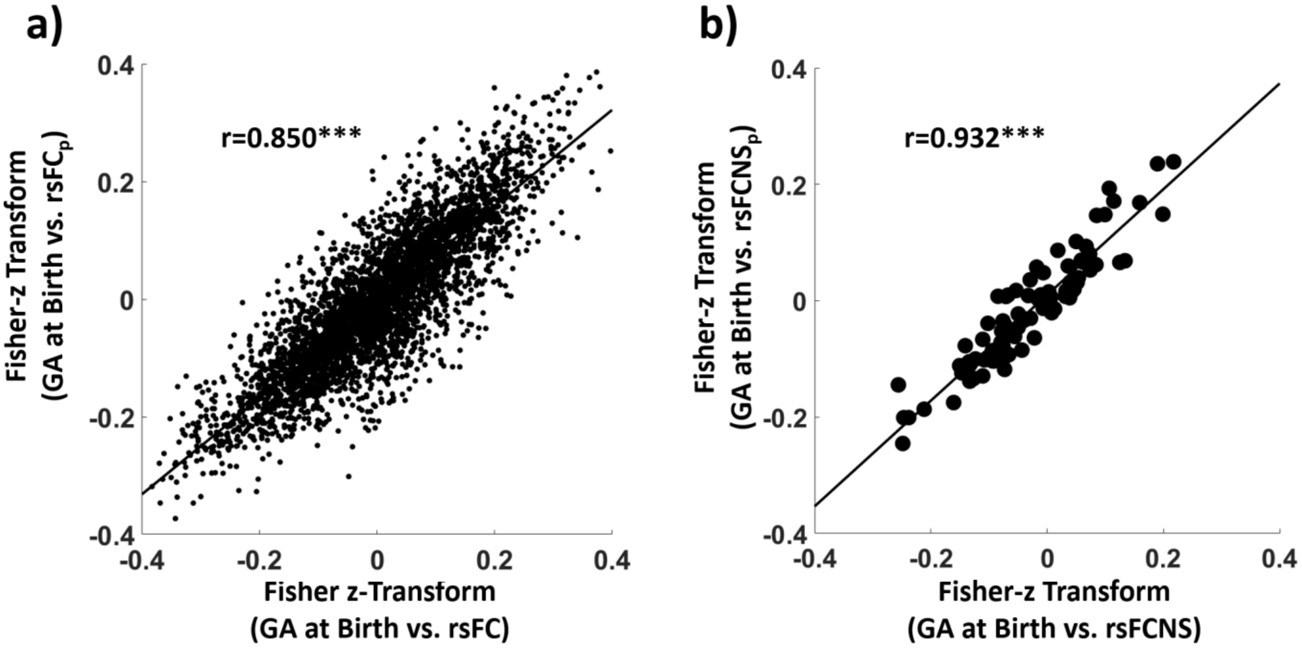

Figure SI8: a) Scatterplot depicting the pairwise spatial associations between the Fisher-z transformed correlation coefficients of rsFC with GA at birth when considering (rsFC) or zeroing out (rsFC$_p$) negative correlations. b) Scatterplot depicting the pairwise spatial associations between the Fisher-z transformed correlation coefficients of rsFCNS with GA at birth when considering (rsFCNS) or zeroing out (rsFCNS$_p$) negative correlations in the rsFC matrix.

Secondly, univariate control analysis was performed to evaluate the effect of motion artifact. An average number of 7 volumes per subject (SD of 5 volumes) was deemed as outliers. The number of

motion outliers and the variance of the 6 motion DVARS signals showed no significant univariate correlations with GA at birth (all r's<0.1, all p's n.s.).

SI3. Results of the Multivariate Analyses (Additional Figures)

Figure SI9 reports the associations between the β-weights of univariate and multivariate regressions for the different metrics considered.

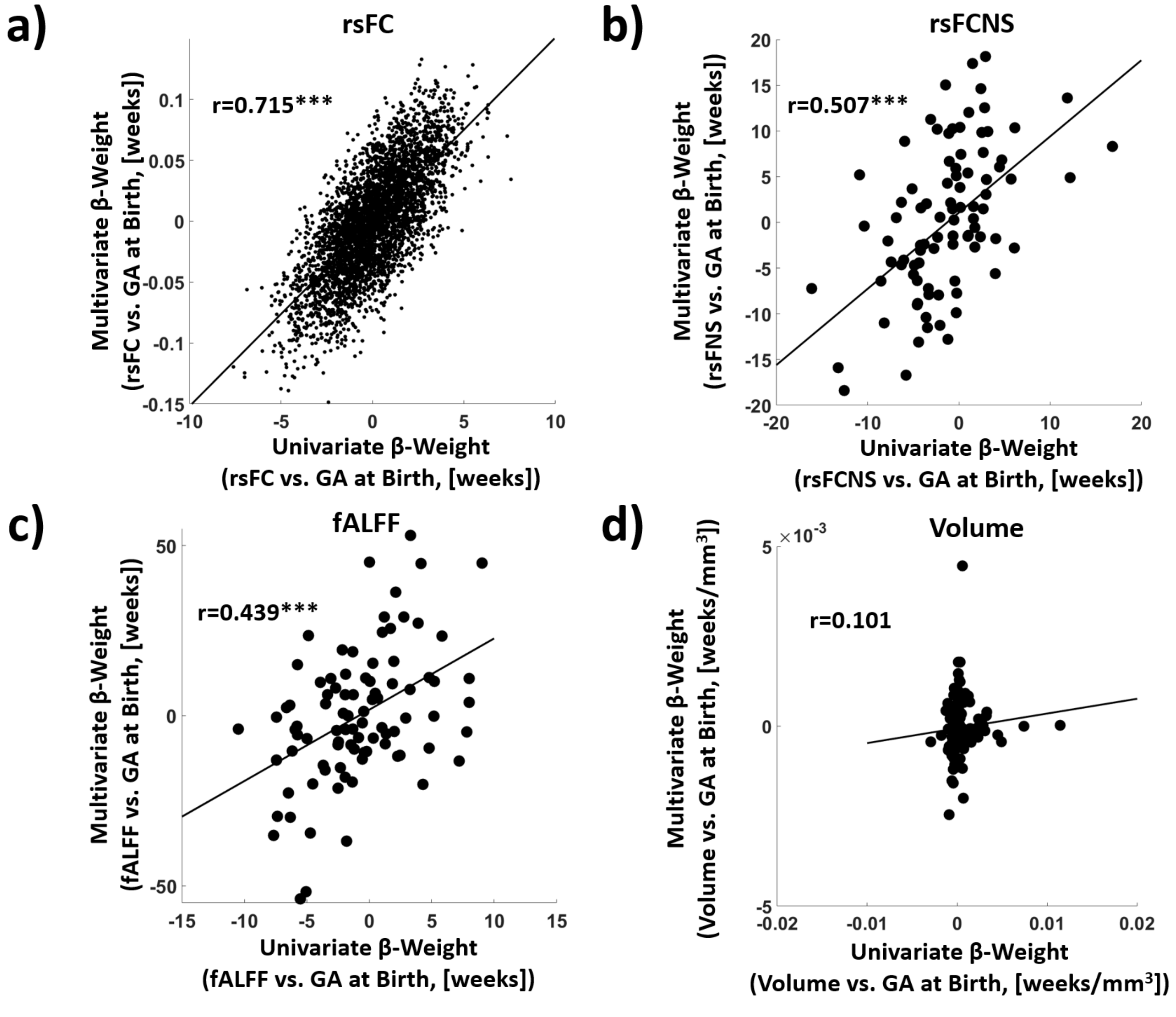

Figure SI9: Scatterplots showing the associations between the β-weights of univariate and multivariate regressions for a) rsFC, b) rsFCNS, c) fALFF and d) regional volume (*** $p<10^{-3}$).

Figure SI10a,b shows the regression and classification outcomes of the 10-fold nCV multivariate analyses on rsFC and rsFCNS when negative correlations were zeroed out. Figure SI10c reports the regression and classification outcomes of the 10-fold nCV multivariate analysis when rsFC, fALFF and Volume were combined to synergistically infer GA at Birth.

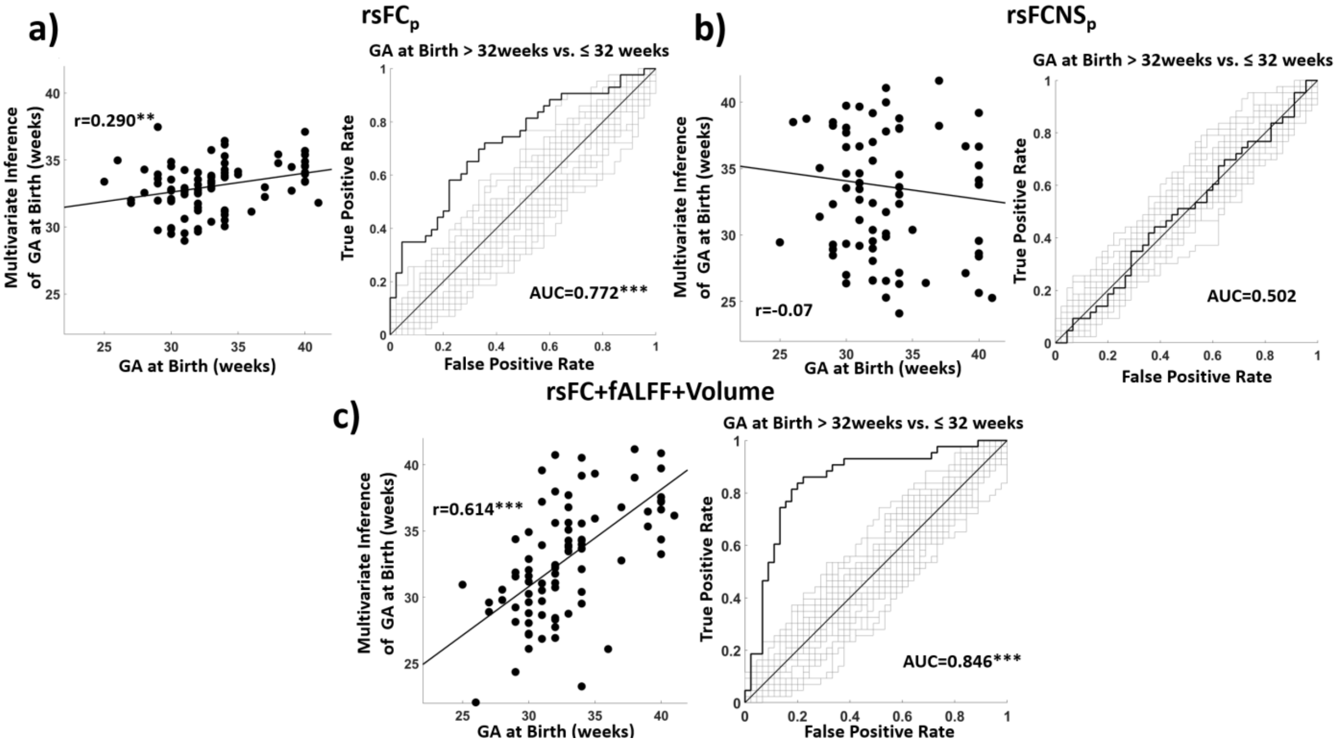

Figure SI10: Generalization outcomes of the 10-fold nCV procedure for the regression (with GA at birth expressed in weeks) and the classification (GA at birth > 32 weeks vs. ≤ 32 weeks) approach. The ROC plots true classification curves (black lines) and curves associated with random shuffled data (gray lines). Outcome of the analysis on a) rsFC considering only positive correlations, rsFC$_p$, b) rsFCNS considering only positive correlations in the rsFC matrix, rsFCNS$_p$, c) combination of rsFC, fALFF and Volume.

SI4. Technical Limitations of the Study

A technical limitation of the study is the use of ROIs, derived from the UNC Infant Atlas, that are defined by anatomical characteristics, i.e., using main sulci as landmarks (Shi, 2011). The rationale behind this choice was to maximize study replicability and comparability across metrics. However, the bias toward anatomical criteria for ROI definition might in part explain the higher performance of

regional volume over other metrics in inferring GA at birth. A possible solution to obtain an unbiased ROI definition would be to work at the level of voxels instead of ROIs, but the advantages of the voxel-level approach need to be contrasted with the increased measurement error and the large number of features that can undermine the sensitivity of univariate and multivariate analyses (Fornito 2010). Moreover, as all the functional metrics were based on standard BOLD imaging, they are affected by known limitations of this technique. In particular, the BOLD signal depends on a non-linear combination of several physiological variables (e.g. cerebral metabolic rate of oxygen, cerebral blood flow and blood volume), and instrumental factors (e.g. field strength and signal to noise ratio) [Sicard and Duong, 2005]. Therefore, functional metrics were evaluated relying on signal filtering and statistical procedures (e.g. correlation analyses) which do not directly quantify the underlying physiology. A limitation of such approaches is the inability to extract instrumentation- or technology-independent quantitative information. This implies that the weak effects found by the univariate analyses might be driven by the diminished sensitivity to the underlying physiology of the metrics employed and, for the multivariate analyses, such data-driven approaches would at least require inter-scanner validation to be used for single subject prediction. An avenue for future investigation is to apply the current multivariate analyses on novel multimodal MRI approaches that extract regional quantitative physiological parameters associated with brain function (e.g. metabolic activity) [Germuska et al., 2019; Lin et al., 2008; Merola et al., 2017; Merola et al., 2018; Niesporek et al., 2018; Qi et al., 2018; Wise et al., 2013].

Finally, it should be acknowledged that the current multivariate analyses were conducted on a number of ROIs-derived features that was generally higher than the number of infants. Especially when features are highly correlated [Magidson, 2013], this disproportion can result in unstable prediction as a function of noise and poor generalization [Liu and Gillies, 2016]. This problem was tackled in the present study by using a space reduction approach, i.e. the PLS, whose efficacy was proven by the

estimated low number of optimal PLS components and the nCV generalization outcome of the regression and classification. However, increasing the sample size would be essential in future studies to improve the performance of the multivariate linear regression but also to explore non-linearities in the data using kernel-based or neural network approaches.

Supplementary Information References

Ball G, Boardman JP, Aljabar P, Pandit A, Arichi T, Merchant N, Rueckert D, Edwards AD, Counsell SJ (2013): The influence of preterm birth on the developing thalamocortical connectome. Cortex 49:1711–1721.

Ball G, Boardman JP, Rueckert D, Aljabar P, Arichi T, Merchant N, Gousias IS, Edwards AD, Counsell SJ (2012): The Effect of Preterm Birth on Thalamic and Cortical Development. Cereb Cortex 22:1016–1024.

Ball G, Pazderova L, Chew A, Tusor N, Merchant N, Arichi T, Allsop JM, Cowan FM, Edwards AD, Counsell SJ (2015): Thalamocortical Connectivity Predicts Cognition in Children Born Preterm. Cereb Cortex 25:4310–4318.

Germuska M, Chandler HL, Stickland RC, Foster C, Fasano F, Okell TW, Steventon J, Tomassini V, Murphy K, Wise RG (2019): Dual-calibrated fMRI measurement of absolute cerebral metabolic rate of oxygen consumption and effective oxygen diffusivity. NeuroImage 184:717–728.

Lin A-L, Fox PT, Yang Y, Lu H, Tan L-H, Gao J-H (2008): Evaluation of MRI models in the measurement of CMRO2 and its relationship with CBF. Magn Reson Med 60:380–389.

Liu R, Gillies DF (2016): Overfitting in linear feature extraction for classification of high-dimensional image data. Pattern Recognit 53:73–86.

Magidson J (2013): Correlated Component Regression: Re-thinking Regression in the Presence of Near Collinearity. In: Abdi, H, Chin, WW, Esposito Vinzi, V, Russolillo, G, Trinchera, L, editors. New Perspectives in Partial Least Squares and Related Methods. New York, NY: Springer. Springer Proceedings in Mathematics & Statistics pp 65–78.

Merola A, Germuska MA, Murphy K, Wise RG (2018): Assessing the repeatability of absolute CMRO2, OEF and haemodynamic measurements from calibrated fMRI. NeuroImage 173:113–126.

Merola A, Germuska MA, Warnert EA, Richmond L, Helme D, Khot S, Murphy K, Rogers PJ, Hall JE, Wise RG (2017): Mapping the pharmacological modulation of brain oxygen metabolism: The effects of caffeine on absolute CMRO2 measured using dual calibrated fMRI. NeuroImage 155:331–343.

Niesporek SC, Umathum R, Lommen JM, Behl NGR, Paech D, Bachert P, Ladd ME, Nagel AM (2018): Reproducibility of CMRO2 determination using dynamic 17O MRI. Magn Reson Med 79:2923–2934.

Qi Y, Liu P, Lin Z, Lu H, Wang X (2018): Hemodynamic and Metabolic Assessment of Neonates With Punctate White Matter Lesions Using Phase-Contrast MRI and T2-Relaxation-Under-Spin-Tagging (TRUST) MRI. Front Physiol 9. https://www.frontiersin.org/articles/10.3389/fphys.2018.00233/full.

Sicard KM, Duong TQ (2005): Effects of hypoxia, hyperoxia, and hypercapnia on baseline and stimulus-evoked BOLD, CBF, and CMRO2 in spontaneously breathing animals. NeuroImage 25:850–858.

Wise RG, Harris AD, Stone AJ, Murphy K (2013): Measurement of OEF and absolute CMRO2: MRI-based methods using interleaved and combined hypercapnia and hyperoxia. NeuroImage 83:135–147.
